# Supplementary material for: Identifying novel prodromal symptoms of eclampsia: A two-country, case-control study
Source: PLoS Med. 2026 Apr 28;23(4):e1004994. doi: 10.1371/journal.pmed.1004994 (PMC13123989; doi:10.1371/journal.pmed.1004994)
Supplement: S1 File — Table A: Symptoms asked in the survey. Table B: Additional participant characteristics (not listed in Table 1 of the main paper) in the pregnancies affected by preeclampsia, eclampsia, or neither (normotensive pregnancies). Table C: Participant characteristics and pregnancy outcomes by study site. Table D: The incidence of various symptoms and the odds of subsequently developing eclampsia – Full list of questions on symptoms screened. Table E: The incidence of various symptoms in the normotensive and eclampsia cohorts, and the odds of subsequently developing eclampsia. Table F: Likelihood of developing eclampsia comparing women recruited with preeclampsia and subsequently developed eclampsia to those with preeclampsia only. Table G: Adjusted odds ratio for the likelihood of developing eclampsia compared to those with preeclampsia. (DOCX) [file pmed.1004994.s001.docx]

**Table A: Symptoms asked in the survey**

| **Symptoms experienced before seizure onset or inclusion** | **Response** |
| --- | --- |
| Edema | None  Hands and/or Feet  Face |
| Did she experience headaches | No  Yes, mild  Yes, moderate  Yes, severe  Yes, worst headache ever experienced |
| Were headaches persistent despite painkillers | No  Yes |
| Headache onset | No headache  Gradual  Sudden |
| Visual disturbances | No visual disturbances  Blurred vision  Double vision  Temporary loss of vision  Sensitivity to light  Partial loss of vision or blind spots in  normal  field of vision  Flickering lights  other |
| If other visual disturbance, please state | Text |
| Time and onset of visual disturbances | No visual disturbances  More than 3 days ago  1-3 days ago  Less than 24 hours ago  Less than 12 hours before  Less than 6 hours before  Less than one hour before |
| Did she experience epigastric or abdominal pain? | No abdominal pain  Yes mild  Yes moderate  Yes severe  Yes worst abdominal pain of my life |
| Tightness in the chest? | No  yes |
| Shortness of breath | No  Yes |
| Focal neurological deficit | None  One extremity  Two extremities  > two extremities  Loss of speech  Facial right side  Facial left side  Severe vertigo  Other |
| Nausea | No  Yes |
| Vomiting | No  Yes |
| Did she have any form of confusion | No  Yes |
| Twitching or jerking of arms and/or legs | No  Yes |
| Diﬃculty concentrating | No  Yes |
| Speech aﬀected | No  Yes |
| Hearing aﬀected | No  Yes |
| Changes in mood | No  Unease  Anger  Depressed  Excited |
| Did she feel anxious? | No  Yes |
| Did she experience a feelings of impending doom or a feeling of the end of the world? | No  Yes |
| Did she experience severe dizziness? | No  Yes |
| Did she experience any weakness or paralysis? | No  Yes |
| Did she experience Jitters /nervousness / nervous  shaking? | No  Yes |

**Table B: Additional participant characteristics (not listed in Table 1 of the main paper) in the pregnancies affected by preeclampsia, eclampsia or neither (normotensive pregnancies).**

| **Characteristic** | **Eclampsia**  **N (%)** | **Preeclampsia**  **N (%)** | **Normotensive**  **N (%)** |
| --- | --- | --- | --- |
| **Proteinuria at booking (%)** |  |  |  |
| Clear | 171 (50.2%) | 934 (68.9%) | 357 (91.8%) |
| Trace | 4 (1.2%) | 41 (3.0%) | 4 (1.0%) |
| 1+ | 11 (3.2%) | 66 (4.9%) | 9 (2.3%) |
| 2+ | 14 (4.1%) | 44 (3.3%) | 2 (0.5%) |
| 3+ | 17 (5.0%) | 30 (2.2%) | 0 (0%) |
| Missing | 124 (36.4%) | 240 (17.7%) | 17 (4.4%) |
| **Complications affecting current pregnancy** | | | |
| Diabetes |  |  |  |
| *Type 1 or 2 diabetes* | 2 (0.6%) | 25 (1.9%) | 0 (0%) |
| *Gestational diabetes* | 9 (2.6%) | 55 (4.1%) | 10 (2.6%) |
| Chronic hypertension | 37 (10.9%) | 218 (16.1%) | 2 (0.5%) |
| Cardiovascular disease | 1 (0.3%) | 10 (0.7%) | 0 (0%) |
| Autoimmune disease | 1 (0.3%) | 6 (0.4%) | 1 (0.3%) |
| Renal disease | 1 (0.3%) | 6 (0.4%) | 1 (0.3%) |
| Neurological disease | 0 (0%) | 4 (0.3%) | 2 (0.5%) |
| **Previous viable pregnancy outcomes** | | | |
| Gestational hypertension | 20 (5.9%) | 106 (7.8%) | 9 (2.3%) |
| Preeclampsia | 17 (5.0%) | 101 (7.5%) | 13 (3.3%) |
| Eclampsia | 15 (4.4%) | 5 (0.4%) | 3 (0.8%) |
| HELLP | 2 (0.6%) | 5 (0.4%) | 2 (0.5%) |
| Placental abruption | 2 (0.6%) | 7 (0.5%) | 14 (3.6%) |
| N/A (First pregnancy) | 211 (61.9%) | 599 (44.2%) | 112 (28.8%) |

**Table C: Participant characteristics and pregnancy outcomes by study site**

| **Characteristic** | **Pakistan (N=632)** | **South Africa (N=1453)** |
| --- | --- | --- |
| **Maternal Characteristics** | | |
| **Maternal age, median (IQR)** | 28 (25.0, 31.7) | 28 (22, 33) |
| Missing | 0 (0%) | 2 (0.1%) |
| **Race (%)** |  |  |
| Black | 0 (0%) | 930 (64.0%) |
| Biracial | 0 (0%) | 513 (35.3%) |
| Caucasian | 0 (0%) | 8 (0.6%) |
| Indian | 0 (0%) | 1 (0.1%) |
| South Asia | 632 (100%) | 1 (0.1%) |
| **Study Group (%)** |  |  |
| Eclampsia | 183 (29.0%) | 158 (10.9%) |
| Preeclampsia | 408 (64.6%) | 947 (65.2%) |
| Normotensive | 41 (6.5%) | 348 (24.0%) |
| **Body mass index (BMI), mean (SD)** | 28.0 (5.6) | 29.3 (7.8) |
| Missing | 1 (0.2%) | 123 (8.5%) |
| **Gestation at inclusion (week; median (IQR))** | 35.4 (32.7, 37.6) | 34.4 (30.1, 37.7) |
| Missing | 27 (10.3%) | 88 (6.1%) |
| **Parity (%)** |  |  |
| Nulliparous | 340 (53.8%) | 582 (40.1%) |
| Multiparous | 291 (46.0%) | 871 (59.9%) |
| Missing | 1 (0.2%) | 0 (0%) |
| **Antenatal care (%)** |  |  |
| None | 274 (43.4%) | 96 (6.6%) |
| After 20 weeks | 184 (29.1%) | 381 (26.2%) |
| Before 20 weeks | 173 (27.4%) | 960 (66.1%) |
| Missing | 1 (0.2%) | 16 (1.1%) |
| **Systolic blood pressure at booking (median (IQR))** | 120 (110, 140) | 122 (113, 131) |
| Missing | 275 (43.5%) | 116 (8.0%) |
| **Diastolic blood pressure at booking (median (IQR))** | 80 (70, 90) | 72 (64, 80) |
| Missing | 275 (43.5%) | 115 (7.9%) |
| **Anti-hypertensive treatments at booking (%)** | 120 (19.0%) | 53 (3.7%) |
| **Tobacco use (%)** | 1 (0.2%) | 155 (10.7%) |
| **Proteinuria at booking (%)** |  |  |
| Clear | 250 (39.6%) | 1212 (83.4%) |
| Trace | 10 (1.6%) | 39 (2.7%) |
| 1+ | 29 (4.6%) | 57 (3.9%) |
| 2+ | 37 (5.9%) | 23 (1.6%) |
| 3+ | 31 (4.9%) | 16 (1.1%) |
| Missing | 275 43.5%) | 106 (7.3%) |
| **Complications affecting current pregnancy** | | |
| Diabetes |  |  |
| *Pre-existing* | 17 (2.7%) | 10 (0.7%) |
| *Gestational diabetes* | 65 (10.3%) | 9 (0.6%) |
| Chronic hypertension | 136 (21.5%) | 121 (8.3%) |
| Cardiovascular disease | 9 (1.4%) | 2 (0.1%) |
| Autoimmune disease | 8 (1.3%) | 0 (0.0%) |
| Renal disease | 8 (1.3%) | 0 (0.0%) |
| Neurological disease | 2 (0.3%) | 4 (0.3%) |
| **Neonatal Characteristics** | | |
| **Stillborn (%)**  Missing | 65 (10.3%)  4 (0.6%) | 115 (7.9%)  98 (6.7%) |
| **Fetal sex (%)** |  |  |
| Female | 311 (49.2%) | 673 (46.3%) |
| Missing | 4 (0.6%) | 137 (9.4%) |
| **Birthweight (median (IQR))** | 2130 (1600, 2800) | 2305.5 (1455, 2980) |
| Missing | 12 (1.9%) | 139 (9.6%) |
| **Gestational age at birth (median (IQR))** | 35.7 (33.3, 37.7) | 35.7 (32.0, 38.4) |
| Missing | 147 (23.3%) | 70 (4.8%) |
| **Mode of birth (%)^** |  |  |
| Vaginal birth | 128 (20.3%) | 491 (33.8%) |
| Cesarean section | 499 (79.1%) | 841 (57.9%) |
| Missing | 4 (0.6%) | 121 (8.3%) |

Materna and neonatal characteristics presented by recruitment site. Continuous data is presented as mean and standard deviation (SD) or median and interquartile range (IQR) based on distribution. Categorical data is presented as frequency and percentages.

^1 case of maternal death antenatally and remained undelivered.

**Table D: The incidence of various symptoms and the odds of subsequently developing eclampsia – Full list of questions on symptoms screened**

| **Variable** | **Pregnancy Status** | | **Univariable analyses** | |
| --- | --- | --- | --- | --- |
|  | **Eclampsia**  **(N=341)** | **Pre-Eclampsia**  **(N=1,355)** | **Odds Ratio (95% confidence intervals)** | ***P*** |
| **Edema (%)** | |  |  |  |
| No | 26 (7.6%) | 164 (12.1%) | Ref | – |
| Yes | 315 (92.4%) | 1,191 (87.9%) | 1.67 (1.08, 2.57) | 0.015 |
| **Headache (%)** | |  |  |  |
| No | 41 (12.0%) | 351 (25.9%) | Ref | – |
| Yes | 300 (88.0%) | 1,004 (74.1%) | 2.56 (1.81, 3.62) | <0.001 |
| **Headache Severity (%)** | |  |  |  |
| No headache | 41 (12.0%) | 351 (25.9%) | Ref | – |
| Mild | 44 (12.9%) | 478 (35.3%) | 0.79 (0.50, 1.23) | 0.30 |
| Moderate | 63 (18.5%) | 228 (16.8%) | 2.37 (1.54, 3.63) | <0.001 |
| Severe | 118 (34.3%) | 224 (16.5%) | 4.47 (3.02, 6.63) | <0.001 |
| Worst headache ever experienced | 76 (22.3%) | 74 (5.5%) | 8.79 (5.58, 13.86) | <0.001 |
| **Headache Onset (%)** | |  |  |  |
| No headache | 41 (12.0%) | 351 (25.9%) | Ref | – |
| Gradual | 183 (53.7%) | 876 (64.7%) | 1.79 (1.25, 2.56) | 0.0010 |
| Sudden | 113 (33.1%) | 123 (9.1%) | 7.86 (5.21, 11.88) | <0.001 |
| **Visual Disturbances (%)** | | |  |  |
| No | 119 (34.9%) | 1,022 (75.4%) | Ref | – |
| Yes | 222 (65.1%) | 333 (24.6%) | 5.73 (4.44, 7.39) | <0.001 |
| **Time and onset of visual disturbances, relative to time of assessment (%)** | | | | |
| No visual disturbance | 119 (34.9%) | 1,022 (75.4%) | Ref | – |
| <1 hour before | 62 (18.2%) | 75 (5.5%) | 7.10 (4.82, 10.45) | <0.001 |
| < 6 hours before | 45 (13.2%) | 28 (2.1%) | 13.80 (8.30, 22.95) | <0.001 |
| < 12 hours before | 43 (12.6%) | 60 (4.4%) | 6.15 (3.98, 9.51) | <0.001 |
| < 24 hours before | 32 (11.1%) | 64 (4.7%) | 5.10 (3.27, 7.95) | <0.001 |
| 1-3 days before | 28 (8.2%) | 90 (6.6%) | 2.67 (1.68, 4.25) | <0.001 |
| ≥ 3 days before | 6 (1.8%) | 16 (1.2%) | 3.22 (1.24, 8.39) | 0.029 |
| **Epigastric pain (%)** | |  |  |  |
| No | 198 (58.1%) | 1,026 (75.7%) | Ref | – |
| Yes | 143 (41.9%) | 329 (24.3%) | 2.25 (1.76, 2.89) | <0.001 |
| **Epigastric or abdominal pain severity (%)** | | |  |  |
| No | 198 (58.1%) | 1,026 (75.7%) | Ref | – |
| Mild | 62 (18.2%) | 201 (14.8%) | 1.60 (1.16, 2.21) | 0.0060 |
| Moderate | 58 (17.0%) | 93 (6.9%) | 3.23 (2.25, 4.64) | <0.001 |
| Severe | 16 (4.7%) | 26 (1.9%) | 3.19 (1.68, 6.05) | 0.0010 |
| Worst abdominal pain of my life | 7 (2.1%) | 9 (0.7%) | 4.03 (1.48, 10.95) | 0.010 |
| **Twitching or jerking of arms and/or legs (%)** | | | |  |
| No | 237 (69.5%) | 1,341 (99.0%) | Ref | – |
| Yes | 104 (30.5%) | 14 (1.0%) | 42.03 (23.66, 74.68) | <0.001 |
| **Hearing affected** | |  |  |  |
| No | 269 (78.9%) | 1,345 (99.3%) | Ref | – |
| Yes | 72 (21.1%) | 10 (0.7%) | 36.00 (18.34, 70.65) | <0.001 |
| **Mind state affected (%)** |  |  |  |  |
| No | 209 (61.3%) | 1,330 (98.2%) | Ref | – |
| Yes | 132 (38.7%) | 25 (1.8%) | 33.60 (21.39, 52.78) | <0.001 |
| **Speech affected (%)** | |  |  |  |
| No | 240 (70.4%) | 1,338 (98.8%) | Ref | – |
| Yes | 101 (29.6%) | 17 (1.2%) | 33.12 (19.46, 54.37) | <0.001 |
| **Severe vertigo (%)** |  |  |  |  |
| No | 322 (94.4%) | 1,352 (99.8%) | Ref | – |
| Yes | 19 (5.6%) | 3 (0.2%) | 26.59 (7.82, 90.41) | <0.001 |
| **Feelings of impending doom or the end of the world (%)** | | | |  |
| No | 186 (54.6%) | 1,309 (96.6%) | Ref | – |
| Yes | 155 (45.4%) | 46 (3.4%) | 23.71 (16.49, 34.10) | <0.001 |
| **Confusion (%)** |  |  |  |  |
| No | 200 (58.7%) | 1,310 (96.7%) | Ref | – |
| Yes | 141 (41.3%) | 45 (3.3%) | 20.52 (14.22, 29.63) | <0.001 |
| **Jitters / nervousness / nervous shaking (%)** | | |  |  |
| No | 271 (79.5%) | 1,336 (98.6%) | Ref | – |
| Yes | 70 (20.5%) | 19 (1.4%) | 18.16 (10.76, 30.66) | <0.001 |
| **Difficulty concentrating (%)** | |  |  |  |
| No | 149 (43.7%) | 1,249 (92.2%) | Ref | – |
| Yes | 192 (56.3%) | 106 (7.8%) | 15.18 (11.34, 20.33) | <0.001 |
| **Weakness or paralysis (%)** | |  |  |  |
| No | 272 (79.8%) | 1,323 (97.6%) | Ref | – |
| Yes | 69 (20.2%) | 32 (2.4%) | 10.49 (6.76, 16.27) | <0.001 |
| **Severe dizziness (%)** | |  |  |  |
| No | 184 (54.0%) | 1,198 (88.4%) | Ref | – |
| Yes | 157 (46.0%) | 157 (11.6%) | 6.51 (4.97, 8.53) | <0.001 |
| **Feel anxious (%)** |  |  |  |  |
| No | 164 (48.1%) | 1,141 (84.2%) | Ref | – |
| Yes | 177 (51.9%) | 214 (15.8%) | 5.75 (4.45, 7.45) | <0.001 |
| **Changes in mood – Any (%)** | | |  |  |
| No | 96 (28.2%) | 953 (70.3%) | Ref | – |
| Yes | 245 (71.8%) | 402 (29.7%) | 6.05 (4.65, 7.87) | <0.001 |
| **Changes in mood – Unease (%)** | | |  |  |
| No | 168 (49.3%) | 1,111 (82.0%) | Ref | – |
| Yes | 173 (50.7%) | 244 (18.0%) | 4.69 (3.64, 6.04) | <0.001 |
| **Changes in mood – Anger (%)** | |  |  |  |
| No | 297 (87.1%) | 1,225 (90.4%) | Ref | – |
| Yes | 44 (12.9%) | 130 (9.6%) | 1.40 (0.97, 2.01) | 0.079 |
| **Changes in mood – Depression (%)** | | |  |  |
| No | 314 (92.1%) | 1,327 (97.9%) | Ref | – |
| Yes | 27 (7.9%) | 28 (2.1%) | 4.08 (2.37, 7.01) | <0.001 |
| **Changes in mood – Excitement (%)** | | |  |  |
| No | 339 (99.4%) | 1,352 (99.8%) | Ref | – |
| Yes | 2 (0.6%) | 3 (0.2%) | 2.66 (0.44, 15.98) | 0.31 |
| **Changes in mood – Other (%)** | |  |  |  |
| No | 337 (98.8%) | 1,347 (99.4%) | Ref | – |
| Yes | 4 (1.2%) | 8 (0.6%) | 2.00 (0.60, 6.68) | 0.28 |
| **Focal neurological deficit (excluding vertigo) (%)** | | | | |
| No | 329 (94.5%) | 1,339 (98.8%) | Ref | – |
| Yes | 12 (3.5%) | 16 (1.2%) | 3.05 (1.43, 6.51) | 0.0060 |
| **Tightness in the chest (%)** | |  |  |  |
| No | 279 (81.8%) | 1,203 (88.8%) | Ref | – |
| Yes | 62 (18.2%) | 152 (11.2%) | 1.76 (1.27, 2.43) | 0.0010 |
| **Shortness of breath (%)** | |  |  |  |
| No | 269 (78.9%) | 1,146 (84.6%) | Ref | – |
| Yes | 72 (21.1%) | 209 (15.4%) | 1.47 (1.09, 1.98) | 0.014 |
| **Nausea (%)** |  |  |  |  |
| No | 214 (62.8%) | 1,081 (79.8%) | Ref | – |
| Yes | 127 (37.2%) | 274 (20.2%) | 2.34 (1.81, 3.03) | <0.001 |
| **Vomiting (%)** |  |  |  |  |
| No | 243 (72.3%) | 1,183 (87.3%) | Ref | – |
| Yes | 98 (28.7%) | 172 (12.7%) | 2.77 (2.09, 3.68) | <0.001 |

Odds ratios and corresponding 95% confidence intervals were estimated via univariable logistic regression for the presence of symptoms being present prior to the onset of eclampsia compared to being present among women with preeclampsia.

**Table E: The incidence of various symptoms in the normotensive and eclampsia cohorts, and the odds of subsequently developing eclampsia.**

| **Variable** | **Pregnancy Status** | | **Univariable analyses** | |
| --- | --- | --- | --- | --- |
|  | **Eclampsia**  **(n=341)** | **Normotensive**  **(n=389)** | **Odds Ratio (95% confidence interval)** | ***P*** |
| **Edema (%)** | |  |  |  |
| No | 26 (7.6%) | 351 (90.2%) | Ref | – |
| Yes | 315 (92.4%) | 38 (9.8%) | 111.91 (66.43, 118.52) | <0.001 |
| **Headache (%)** | |  |  |  |
| No | 41 (12.0%) | 376 (96.7%) | Ref | – |
| Yes | 300 (88.0%) | 13 (3.3) | 211.63 (111.36, 402.18) | <0.001 |
| **Headache Severity (%)** | |  |  |  |
| No headache | 41 (12.0%) | 376 (96.7%) | Ref | – |
| Mild | 44 (12.9%) | 5 (1.3%) | 80.70 (30.30, 214.93) | <0.001 |
| Moderate | 63 (18.5%) | 5 (1.3%) | 115.55 (43.98, 303.60) | <0.001 |
| Severe | 118 (34.3%) | 3 (0.8%) | 357.66 (108.76, 1176.21) | <0.001 |
| Worst headache ever experienced | 76 (22.3%) | 0 (0%) | – | – |
| **Headache Onset (%)** | |  |  |  |
| No headache | 41 (12.0%) | 376 (96.7%) | Ref | – |
| Gradual | 183 (53.7%) | 10 (2.6%) | 167.82 (82.22, 342.54) | <0.001 |
| Sudden | 113 (33.1%) | 3 (0.8%) | 345.43 (105.00, 1136.55) | <0.001 |
| **Visual Disturbances (%)** | | |  |  |
| No | 119 (34.9%) | 387 (99.5%) | Ref | – |
| Yes | 222 (65.1%) | 2 (0.5%) | 360.98 (88.38, 1474.45) | <0.001 |
| **Epigastric pain (%)** | |  |  |  |
| No | 198 (58.1%) | 385 (99.0%) | Ref | – |
| Yes | 143 (41.9%) | 4 (1.0%) | 69.51 (25.36, 190.53%) | <0.001 |
| **Twitching or jerking of arms and/or legs (%)** | | | |  |
| No | 237 (69.5%) | 388 (99.7%) | Ref | – |
| Yes | 104 (30.5%) | 1 (0.3%) | 170.26 (23.60, 1228.21) | <0.001 |
| **Hearing affected (%)** | |  |  |  |
| No | 269 (79.9%) | 389 (100.0%) | Ref | – |
| Yes | 72 (21.1%) | 0 (0%) | – | – |
| **Mind state affected (%)** |  |  |  |  |
| No | 209 (61.3%) | 389 (100.0%) | Ref | – |
| Yes | 132 (38.7%) | 0 (0%) | – | – |
| **Speech affected (%)** | |  |  |  |
| No | 240 (70.4%) | 389 (100.0%) | Ref | – |
| Yes | 101 (29.6%) | 0 (0%) | – | – |
| **Severe vertigo (%)** |  |  |  |  |
| No | 322 (94.4%) | 388 (99.7%) | Ref | – |
| Yes | 19 (5.6%) | 1 (0.3%) | 22.89 (3.05, 171.95) | <0.001 |
| **Feelings of impending doom or the end of the world (%)** | | | |  |
| No | 186 (54.6%) | 389 (100.0%) | Ref | – |
| Yes | 155 (45.4%) | 0 (0%) | – | – |
| **Confusion (%)** |  |  |  |  |
| No | 200 (58.7%) | 388 (99.7%) | Ref | – |
| Yes | 141 (41.3%) | 1 (0.3%) | 273.54 (37.98, 1969.90) | <0.001 |
| **Jitters / nervousness / nervous shaking (%)** | | |  |  |
| No | 271 (79.5%) | 389 (100.0%) | Ref | – |
| Yes | 70 (20.5%) | 0 (0%) | – | – |
| **Difficulty concentrating (%)** | |  |  |  |
| No | 149 (43.7%) | 389 (100.0%) | Ref | – |
| Yes | 192 (56.3%) | 0 (0%) | – | – |
| **Weakness or paralysis (%)** | |  |  |  |
| No | 272 (79.8%) | 389 (100.0%) | Ref | – |
| Yes | 69 (20.2%) | 0 (0%) | – | – |
| **Severe dizziness (%)** | |  |  |  |
| No | 184 (54.0%) | 387 (99.5%) | Ref | – |
| Yes | 157 (46.0%) | 2 (0.5%) | 165.11 (40.48, 673.37) | <0.001 |
| **Feel anxious (%)** |  |  |  |  |
| No | 164 (48.1%) | 387 (99.5%) | Ref | – |
| Yes | 177 (51.9%) | 2 (0.5%) | 208.84 (51.21, 851.66) | <0.001 |
| **Changes in mood – Any (%)** | | |  |  |
| No | 96 (28.2%) | 364 (93.6%) | Ref | – |
| Yes | 245 (71.8%) | 25 (6.4%) | 37.16 (23.25, 59.39) | <0.001 |
| **Changes in mood – Unease (%)** | | |  |  |
| No | 168 (49.3%) | 375 (96.4%) | Ref | – |
| Yes | 173 (50.7%) | 14 (3.6%) | 27.58 (15.53, 48.98) | <0.001 |
| **Changes in mood – Anger (%)** | |  |  |  |
| No | 297 (87.1%) | 386 (99.2%) | Ref | – |
| Yes | 44 (12.9%) | 3 (0.8%) | 19.06 (5.86, 61.99) | <0.001 |
| **Changes in mood – Depression (%)** | | |  |  |
| No | 314 (92.1%) | 381 (97.9%) | Ref | – |
| Yes | 27 (7.9%) | 8 (2.1%) | 4.10 (1.83, 9.14) | <0.001 |
| **Changes in mood – Excitement (%)** | | |  |  |
| No | 339 (99.4%) | 389 (100.0%) | Ref | – |
| Yes | 2 (0.6%) | 0 (0%) | – | – |
| **Changes in mood – Other (%)** | |  |  |  |
| No | 337 (98.8%) | 388 (99.7%) | Ref | – |
| Yes | 4 (1.2%) | 1 (0.3%) | 4.61 (0.51, 41.40) | 0.13 |
| **Focal neurological deficit (excluding vertigo) (%)** | | | | |
| No | 329 (96.5%) | 387 (99.5%) | Ref | – |
| Yes | 12 (3.5%) | 2 (0.5%) | 7.06 (1.57, 31.76) | 0.0020 |
| **Tightness in the chest (%)** | |  |  |  |
| No | 279 (81.8%) | 389 (100.0%) | Ref | – |
| Yes | 62 (18.2%) | 0 (0%) | – | – |
| **Shortness of breath (%)** | |  |  |  |
| No | 269 (78.9%) | 389 (100.0%) | Ref | – |
| Yes | 72 (21.1%) | 0 (0%) | – | – |
| **Nausea (%)** |  |  |  |  |
| No | 214 (62.8%) | 387 (99.5%) | Ref | – |
| Yes | 127 (37.2%) | 2 (0.5%) | 114.83 (28.13, 468.81) | <0.001 |
| **Vomiting (%)** |  |  |  |  |
| No | 243 (71.3%) | 388 (99.7%) | Ref | – |
| Yes | 98 (28.7%) | 1 (0.3%) | 156.48 (21.68, 1129.31) | <0.001 |

Odds ratios and corresponding 95% confidence intervals were estimated via univariable logistic regression for the presence of symptoms being present prior to the onset of eclampsia compared to being present among normotensive women.

A dash (-) is shown when an odds ratio cannot be calculated.

**Table F: Likelihood of developing eclampsia comparing women recruited with preeclampsia and subsequently developed eclampsia to those with preeclampsia only.**

| **Variable** | **Pregnancy Status** | | **Univariable analyses** | |
| --- | --- | --- | --- | --- |
|  | **Eclampsia**  **(n=23)** | **Pre-Eclampsia**  **(=1,355)** | **Odds Ratio (95% CI)** | ***P*** |
| **Edema (%)** | |  |  |  |
| No | 5 (21.7) | 164 (12.1%) | Ref | – |
| Yes | 18 (78.3%) | 1,191 (87.9%) | 0.50 (0.18, 1.35) | 0.171 |
| **Headache (%)** | |  |  |  |
| No | 4 (17.4%) | 351 (25.9%) | Ref | – |
| Yes | 19 (82.6%) | 1,004 (74.1%) | 1.66 (0.56, 4.91) | 0.360 |
| **Visual Disturbances (%)** | | |  |  |
| No | 17 (73.9%) | 1,022 (75.4%) | Ref | – |
| Yes | 6 (26.1%) | 333 (24.6%) | 1.08 (0.42, 2.77) | 0.867 |
| **Epigastric pain (%)** | |  |  |  |
| No | 18 (78.3%) | 1,026 (75.7%) | Ref | – |
| Yes | 5 (21.7%) | 329 (24.3%) | 0.87 (0.32, 2.35) | 0.78 |
| **Twitching or jerking of arms and/or legs (%)** | | | |  |
| No | 19 (82.6%) | 1,341 (99.0%) | Ref | – |
| Yes | 4 (17.9%) | 14 (1.0%) | 20.17 (6.07, 66.95) | <0.001 |
| **Hearing affected** | |  |  |  |
| No | 23 (100%) | 1,345 (99.3%) | Ref | – |
| Yes | 0 | 10 (0.7%) | - | - |
| **Mind state affected (%)** |  |  |  |  |
| No | 19 (92.6) | 1,330 (98.2%) | Ref | – |
| Yes | 4 (17.4) | 25 (1.8%) | 11.20 (3.56, 35.32) | <0.001 |
| **Speech affected (%)** | |  |  |  |
| No | 23 (100%) | 1,338 (98.8%) | Ref | – |
| Yes | 0 | 17 (1.2%) | - | - |
| **Severe vertigo (%)** |  |  |  |  |
| No | 23 (100%) | 1,352 (99.8%) | Ref | – |
| Yes | 0 | 3 (0.2%) | - | - |
| **Feelings of impending doom or the end of the world (%)** | | | |  |
| No | 20 (87.0%) | 1,309 (96.6%) | Ref | – |
| Yes | 3 (13.0%) | 46 (3.4%) | 4.27 (1.22, 14.88) | 0.023 |
| **Confusion (%)** |  |  |  |  |
| No | 17 (73.9%) | 1,310 (96.7%) | Ref | – |
| Yes | 6 (26.1%) | 45 (3.3%) | 10.27 (3.87, 27.29) | <0.001 |
| **Jitters / nervousness / nervous shaking (%)** | | |  |  |
| No | 21 (91.3%) | 1,336 (98.6%) | Ref | – |
| Yes | 2 (8.7%) | 19 (1.4%) | 6.70 (1.47, 30.61) | 0.014 |
| **Difficulty concentrating (%)** | |  |  |  |
| No | 20 (87.0%) | 1,249 (92.2%) | Ref | – |
| Yes | 3 (13.0%) | 106 (7.8%) | 1.77 (0.52, 6.04) | 0.364 |
| **Weakness or paralysis (%)** | |  |  |  |
| No | 22 (95.7%) | 1,323 (97.6%) | Ref | – |
| Yes | 1 (4.4%) | 32 (2.4%) | 1.88 (0.25, 14.37) | 0.543 |
| **Severe dizziness (%)** | |  |  |  |
| No | 18 (78.3%) | 1,198 (88.4%) | Ref | – |
| Yes | 5 (21.7) | 157 (11.6%) | 2.12 (0.78, 5.79) | 0.143 |
| **Feel anxious (%)** |  |  |  |  |
| No | 16 (69.6%) | 1,141 (84.2%) | Ref | – |
| Yes | 7 (30.4%) | 214 (15.8%) | 2.33 (0.95, 5.74) | 0.065 |
| **Changes in mood – Any (%)** | | |  |  |
| No | 11 (47.8%) | 953 (70.3%) | Ref | – |
| Yes | 12 (52.2%) | 402 (29.7%) | 2.59 (1.13, 5.91) | 0.024 |
| **Focal neurological deficit (excluding vertigo) (%)** | | | | |
| No | 23 (100%) | 1,339 (98.8%) | Ref | – |
| Yes | 0 | 16 (1.2%) | - | - |
| **Tightness in the chest (%)** | |  |  |  |
| No | 22 (95.7%) | 1,203 (88.8%) | Ref | – |
| Yes | 1 (4.4%) | 152 (11.2%) | 0.36 (0.05, 2.69) | 0.319 |
| **Shortness of breath (%)** | |  |  |  |
| No | 21 (91.3%) | 1,146 (84.6%) | Ref | – |
| Yes | 2 (8.7%) | 209 (15.4%) | 0.52 (1.12, 2.24) | 0.382 |
| **Nausea (%)** |  |  |  |  |
| No | 9 (39.3) | 1,081 (79.8%) | Ref | – |
| Yes | 14 (60.9%) | 274 (20.2%) | 6.14 (2.63, 14.33) | <0.001 |
| **Vomiting (%)** |  |  |  |  |
| No | 15 (65.2) | 1,183 (87.3%) | Ref | – |
| Yes | 8 (34.8%) | 172 (12.7%) | 3.67 (1.53, 8.78) | 0.004 |

Odds ratios and corresponding 95% confidence intervals were estimated via univariable logistic regression for the presence of symptoms being present prior to the onset of eclampsia compared to being present among women who were recruited with preeclampsia and subsequently developed eclampsia (after completing the questionnaire).

**Table G: Adjusted odds ratio for the likelihood of developing eclampsia compared to those with preeclampsia.**

| Symptom | Eclampsia  (N=341) | | Preeclampsia (N=1,355) | Odds Ratio  (95% CI) | Adjusted Odds Ratio  (95% confidence interval)* |
| --- | --- | --- | --- | --- | --- |
| Known prodromal symptoms – N (%) | | | | | |
| Headache | | 300 (88.0) | 1004 (74.1) | 2.56 (1.81 to 3.62) | 3.31 (2.25 to 4.89) |
| Visual disturbances | | 222 (65.1) | 333 (24.6) | 5.73 (4.44 to 7.39) | 6.32 (4.75 to 8.41) |
| Epigastric pain | | 143 (41.9) | 329 (24.3) | 2.25 (1.76 to 2.89) | 1.79 (1.36 to 2.6) |
| New prodromal symptoms – N (%) | | | | | |
| Twitching or jerking of arms or legs | | 105 (30.5) | 14 (1.0) | 42.03 (23.66 to 74.68) | 35.63 (19.37 to 65.54) |
| Hearing affected | | 72 (21.1) | 10 (0.7) | 36.00 (18.34 to 70.65) | 40.98 (18.85 to 89.12) |
| Mind state affected | | 132 (38.7) | 25 (1.8) | 33.60 (21.39 to 52.78) | 39.93 (24.05 to 66.28) |
| Speech affected | | 101 (29.6) | 17 (1.2) | 33.12 (19.46 to 54.37) | 29.44 (16.73 to 51.78) |
| Severe vertigo | | 19 (5.6) | 3 (0.2) | 26.59 (7.82 to 90.41) | 22.11 (6.18 to 79.09) |
| Feelings of impending doom or end of the world | | 155 (45.4) | 46 (3.4) | 23.71 (16.49 to 34.10) | 24.72 (16.32 to 37.47) |
| Confusion | | 141 (41.3) | 45 (3.3) | 20.52 (14.22 to 29.63) | 16.90 (11.40 to 25.06) |
| Jitters/nervousness/nervous shaking | | 70 (20.5) | 19 (1.4) | 18.16 (10.76 to 30.66) | 14.10 (8.04 to 24.74) |
| Difficulty concentrating | | 192 (56.3) | 106 (7.8) | 15.18 (11.34 to 20.33) | 16.11 (11.57 to 22.42) |
| Weakness or paralysis | | 69 (20.2) | 32 (2.4) | 10.49 (6.76 to 16.27) | 9.33 (5.77 to 15.10) |

Odds ratios and corresponding 95% confidence intervals were estimated via multivariable logistic regression for the presence of symptoms being present prior to the onset of eclampsia compared to being present among women with preeclampsia. *Variables included in the multivariable model were maternal age, body mass index and country of recruitment. N=1594 in adjusted analyses
