# Supplementary material for: Identifying novel prodromal symptoms of eclampsia: A two-country, case-control study
Source: PLoS Med. 2026 Apr 28;23(4):e1004994. doi: 10.1371/journal.pmed.1004994 (PMC13123989; doi:10.1371/journal.pmed.1004994)
Supplement: S1 Protocol — (DOCX) [file pmed.1004994.s002.docx]

|  | Improved Characterisation of Eclampsia to optimise maternal and perinatal outcomes: the ICE study |
| --- | --- |

# Study protocol

| Coordinating centre trials office | The University of Melbourne – Department of Obstetrics and Gynaecology, Mercy Hospital for Women, 163 Studley Road, Heidelberg, Victoria, Australia |
| --- | --- |
| Investigational sites: | Tygerberg Hospital, Cape Town, South Africa  Fatima Memorial Hospital, Lahore, Pakistan  Jinnah Hospital, Lahore, Pakistan |
| Principal Investigators | Dr Roxanne Hastie, Dr Cathy Cluver, Dr Lina Bergman, Professor Stephen Tong, Professor Sue Walker |
| Protocol Version Number | Version 6 |

A prospective multicentre observational trial

Protocol authors: Dr Roxanne Hastie, Dr Cathy Cluver, Dr Lina Bergman, Professor Stephen Tong.

## Background

Eclampsia is a serious pregnancy complication that occurs when a pregnant woman - or new mother - experiences seizures associated with hypertension. These seizures pose a major risk to the life and the health of mothers and unborn babies. Signs or symptoms of severe preeclampsia are not always present or easy to recognise before a woman develops eclampsia. This is why it can be difficult to tell which women are likely to develop eclampsia before the first seizure occurs. If we can identify the features that are most common and unique to eclampsia, these could be used to develop a tool to help identify the women displaying signs or symptoms that occur prior to the onset of eclamptic seizures. This would mean that treatment to prevent seizures could be targeted to the right women and quickly administered before they become extremely unwell with this life-threatening complication.

The development of a tool to predict eclampsia in high-resource settings (such as Australia) has been largely hampered by the extremely low prevalence of this complication1. This makes it challenging for researchers in this setting to conduct impactful studies and obtain reliable and detailed information about the characteristic features of eclampsia. However, rates of eclampsia, like many other complications, are far greater in low to middle income countries (LMICs; including South Africa, Pakistan and the Solomon Islands), with an estimated 16-69 cases of eclampsia per 10,000 livebirths2,3. Thus, research conducted in LMIC settings allow for the prospective recruitment of a relatively large cohort of women with eclampsia. They are a useful target population to prospectively characterise the signs and symptoms preceding the disease. *Such characterisation may yield a unique clinical signature for eclampsia and the development of a predictive algorithm.*

## Aims

**Aim 1:** To characterize the clinical signs and symptoms that occur prior to the onset of eclampsia in a prospectively collected cohort.

**Aim 2:** Compare these characteristics to women with preeclampsia and healthy normal pregnancies to identify which features represent a unique clinical signature for eclampsia to form a simple predictive algorithm.

## Study design

This is a multicentre prospective observational study in which women will be consecutively recruited, with four centres currently recruiting:

- Tygerberg Hospital, Cape Town South Africa
- Fatima Memorial Hospital, Lahore, Pakistan
- Jinnah Hospital, Lahore, Pakistan

Clinical signs and symptoms experienced by the women prior to enrolment in this study will be centrally recorded via the online database Research Electronic Data Capture (REDCap) and used to identify the presence or absence of a unique clinical signature able to predict imminent eclampsia.

Chief investigators at each site will be responsible for training and familiarising recruiting and study staff with the use of the REDCap and online data collection via the supplied iPad.

## Recruitment

Women presenting with eclampsia, preeclampsia or healthy pregnancies will be eligible to participate, with recruitment aiming to enrol *all women with eclampsia or preeclampsia at each site*.

**Inclusion criteria**

*Eclampsia:*

- Diagnosed as new onset generalized tonic colonic seizures or coma in pregnant women or women who have recently given birth.
- Singleton pregnancies.
- All gestational ages, including post-partum eclampsia
- Enrolment to the study must occur within 7 days of an eclamptic episode
- Women must be coherent and able to provide informed consent prior to enrolment

*Preeclampsia:*

- Diagnosed as new onset of hypertension (>140 mmHg systolic or > 90 mmHg diastolic) after the 20th week of gestation and the coexistence of one or more of the following new onset conditions:
  - Proteinuria
  - Other maternal organ evolvement:
    - renal insufficiency (creatinine >90 umol/L)
    - liver involvement (elevated transaminases and/or severe right upper quadrant or epigastric pain)
    - neurological complications
    - haematological complications (thrombocytopenia, DIC, haemolysis)
  - Uteroplacental dysfunction
    - Fetal growth restriction
- All gestations >20 weeks
- Singleton pregnancies
- Have not experience an eclamptic episode

*Normotensive controls:*

- Healthy normotensive pregnant woman recruited from women seeking maternity services during the same or similar time period
- Matched by closest gestation and parity.

**Exclusion criteria**

- Women with multiple pregnancies.
- Women with seizures attributed to a diagnosis other than eclampsia such as; central nervous system infections, a history of seizures or epilepsy, medications and/or illicit drug use.
- Women unable to provide informed consent

## Data collection and management

Once eligible participants are identified, they will be provided with a patient information leaflet and invited to partake in the study. If written informed consent is provided:

1. Participant is enrolled in the study
2. Data will be collected by a trained research nurse or clinician (trained in interview techniques and the use of REDCap)
3. Data will be collected by directly asking the participant a series of standardised structured interview questions and review of medical charts
4. Participants will be followed until postpartum discharge, with maternal and neonatal outcomes recorded.
5. Using an iPad, all data will be entered into the secure online database REDCap, with each site having an individual database, with appropriate local translations.
6. All data will be accessible via the lead site and chief investigator.

## Outcome measures

The primary outcome is the presence or absence of a clinical signature of imminent eclampsia, unique both from women with preeclampsia and healthy normotensive pregnant women. To determine this, we have designed a standardised questionnaire collecting the following information:

| Maternal characteristics | Obstetric/booking information | Signs/symptoms | Eclampsia | Outcome |
| --- | --- | --- | --- | --- |
| First name | Gestation at booking | Highest systolic BP | Date of onset | Delivery date |
| Last name | Date at booking | Highest diastolic BP | Location of onset | Gestation at delivery |
| Maternal age | Estimated due date | Highest proteinuria | Prior magnesium sulphate | Indication for delivery |
| Address | How was due date estimated | Lowest platelet count |  | Mode of delivery |
| Race | Height | Highest AST |  | Live born |
| Marital status | Weight | Lowest haemoglobin |  | Sex |
|  | Antenatal care | Edema |  | Birthweight |
|  | Gravidity/parity | Tendon reflexes |  | Apgar at 5 minutes |
|  | Previous viable pregnancy outcome | Headaches, severity and onset |  | Neonatal outcome |
|  | Systolic BP at booking | Visual disturbances/type and onset |  | Number of days in hospital (maternal) |
|  | Diastolic BP at booking | Epigastric pain |  | Adverse maternal outcome |
|  | Proteinuria at booking | Tightness in chest |  |  |
|  | Antihypertensive medication | Shortness of breath |  |  |
|  | Tobacco/alcohol/drug use | Focal neurological deficit |  |  |
|  | Diabetes | Nausea/vomiting |  |  |
|  | Chronic hypertension | Confusion |  |  |
|  | Cardiovascular disease | Twitching/jerking of extremities |  |  |
|  | Anaemia | Mind state |  |  |
|  | HIV status | Concentration |  |  |
|  | Respiratory disease | Speech |  |  |
|  | Inflammatory bowel disease | Hearing |  |  |
|  | Autoimmune disease | Changes in moods |  |  |
|  | Malaria | Dizziness |  |  |
|  | Renal disease | Weakness or paralysis |  |  |
|  | Neurological disease | Jitters/nervousness |  |  |
|  | Medication use |  |  |  |

## Ethical approval

Each site will be responsible for obtaining local ethical approval for this study, with recruitment only commencing once approval has been obtained and approval notification provided to the central chief investigator. All patients must provide informed written consent prior to enrolment in the study.

## Statistical analysis

Data will be collected and analysed in clinically relevant categories with I2 or Mann-Whitney U tests used to detect difference between groups. We will compare data from women with eclampsia to those with i) preeclampsia and ii) normotensive pregnancies, to determine the unique signs and symptoms that precede eclampsia. Odds ratios will be used to determine strength of association for clinical characteristics significantly associated with eclampsia. We will also attempt to create a predictive score for eclampsia.

1. Knight M. Eclampsia in the United Kingdom 2005. *BJOG: An International Journal of Obstetrics & Gynaecology.* 2007;114(9):1072-1078.

2. Osungbade KO, Ige OK. Public Health Perspectives of Preeclampsia in Developing Countries: Implication for Health System Strengthening. *Journal of Pregnancy.* 2011;2011:481095.

3. Frias AE, Jr., Belfort MA. Post Magpie: how should we be managing severe preeclampsia? *Current opinion in obstetrics & gynecology.* 2003;15(6):489-495.
