# Supplementary material for: Identifying novel prodromal symptoms of eclampsia: A two-country, case-control study
Source: PLoS Med. 2026 Apr 28;23(4):e1004994. doi: 10.1371/journal.pmed.1004994 (PMC13123989; doi:10.1371/journal.pmed.1004994)
Supplement: Attachment — Submitted filename: PMEDICINE-D-25-03006_R1.pdf [file pmed.1004994.s007.pdf]

PLOS Medicine

Identifying novel prodromal symptoms of eclampsia: A multi-national, case-cohort study of prospectively collected data

--Manuscript Draft--

**Manuscript Number:**	PMEDICINE-D-25-03006R1
**Full Title:**	Identifying novel prodromal symptoms of eclampsia: A multi-national, case-cohort study of prospectively collected data
**Short Title:**	Novel prodromal symptoms of eclampsia
**Article Type:**	Research Article
**Abstract:**	Background<br><br>Magnesium sulphate halves the risk of eclampsia. There is no consensus on who to give magnesium sulphate prophylaxis because clinical tools are poor at identifying those at risk. Known prodromal symptoms such as headache, visual disturbance or epigastric pain have modest associations with eclampsia. We set out to identify new prodromal symptoms of eclampsia.<br><br>Methods and findings<br><br>This case-control study prospectively recruited participants in South Africa and Pakistan who had eclampsia, preeclampsia or normotensive pregnancies. We asked whether they experienced 20 neurological symptoms, within 7 days of the seizure for those who had eclampsia. The primary analysis was the likelihood of symptoms occurring before eclampsia, compared to being present with preeclampsia.<br>341 participants were recruited with eclampsia, 1355 with preeclampsia and 389 with normotensive pregnancies. When comparing symptoms among those who had eclampsia versus preeclampsia, the odds ratios (OR) were 2.56 (95%CI 1.81-3.62) for headache, 5.73 (95%CI 4.44, 7.39) for visual disturbances and 2.25 (95%CI 1.76, 2.89) for epigastric pain. We identified ten symptoms with odds ratios over 10 for eclampsia. Odds ratios were 42.03 (95%CI 23.66, 74.68) for twitching/jerking limbs (30.5% eclampsia vs 1% preeclampsia), 33.12 (95%CI 19.46, 54.37) for affected hearing (21.1% vs 0.7%), 33.60 (95%CI 21.39, 52.78) for altered mind state (38.7% vs 1.8%); 33.12 for impaired speech, 23.71 for feelings of doom, 26.59 for severe vertigo, 20.52 for confusion, 18.16 for jitters, 15.81 for difficulty concentrating and 10.49 for weakness/paralysis. These symptoms were rare among normotensive pregnancies. Only 2.4% of women with eclampsia did not experience any prodromal symptoms.<br><br>Conclusions<br><br>Ten prodromal symptoms exhibit far stronger associations with eclampsia than headache, visual disturbances or epigastric pain. Eclampsia is uncommon without any prodromal symptoms. It may be useful to screen these symptoms among women with preeclampsia as part of clinical history taking to guide management. They could help direct magnesium sulphate prophylaxis at those with a higher risk of eclampsia.
**Additional Information:**	
**Question**	**Response**
**Financial Disclosure**<br><br>Enter a financial disclosure statement that describes the sources of funding for the work included in this submission. Review the submission guidelines for detailed requirements. View published research articles from *PLOS Medicine* for specific examples.	Yes

Powered by Editorial Manager® and ProduXion Manager® from Aries Systems Corporation

This statement is required for submission and **will appear in the published article** if the submission is accepted. Please make sure it is accurate.<br><br>**Funded studies**<br>Enter a statement with the following details:<br>• Initials of the authors who received each award<br>• Grant numbers awarded to each author<br>• The full name of each funder<br>• URL of each funder website<br>• Did the sponsors or funders play any role in the study design, data collection and analysis, decision to publish, or preparation of the manuscript?<br><br>Did you receive funding for this work?	
Please add funding details.<br>as follow-up to "**Financial Disclosure**<br><br>Enter a financial disclosure statement that describes the sources of funding for the work included in this submission. Review the submission guidelines for detailed requirements. View published research articles from *PLOS Medicine* for specific examples.<br><br>This statement is required for submission and **will appear in the published article** if the submission is accepted. Please make sure it is accurate.<br><br>**Funded studies**<br>Enter a statement with the following details:<br>• Initials of the authors who received each award<br>• Grant numbers awarded to each author<br>• The full name of each funder<br>• URL of each funder website<br>• Did the sponsors or funders play any role in the study design, data collection and analysis, decision to publish, or preparation of the manuscript?	This study supported via the RANZCOG Women's Health Foundation. RH, ST and were all supported by the National Health and Medical Research Council. LB is a Wallenberg Scholar, supported by the Wallenberg Center for Molecular and Translational Medicine (WCMTM). CC receives salary support from the Mercy Health Foundation. The funders had no role in the design and conduct of the study; collection, management, analysis, and interpretation of the data; preparation, review, or approval of the manuscript; and decision to submit the manuscript for publication.

Did you receive funding for this work?"	
Please select the country of your main research funder (please select carefully as in some cases this is used in fee calculation).<br>as follow-up to "**Financial Disclosure**<br><br>Enter a financial disclosure statement that describes the sources of funding for the work included in this submission. Review the submission guidelines for detailed requirements. View published research articles from *PLOS Medicine* for specific examples.<br><br>This statement is required for submission and **will appear in the published article** if the submission is accepted. Please make sure it is accurate.<br><br>**Funded studies**<br>Enter a statement with the following details:<br>• Initials of the authors who received each award<br>• Grant numbers awarded to each author<br>• The full name of each funder<br>• URL of each funder website<br>• Did the sponsors or funders play any role in the study design, data collection and analysis, decision to publish, or preparation of the manuscript?<br><br>Did you receive funding for this work?"	AUSTRALIA - AU
**Competing Interests**<br><br>Use the instructions below to enter a competing interest statement for this submission. On behalf of all authors, disclose any competing interests that could be perceived to bias this work—acknowledging all financial support and any other relevant financial or non-financial competing interests.<br><br>This statement **will appear in the published article** if the submission is	I have read the Journal's policy and authors of this manuscript have the following compeiting interests: Drs Tong, Cluver and Walker provide consultancy services for Diamedica Therapeutics. Drs Cluver and Tong provide consultancy services for Avilar Therapeutics.

Powered by Editorial Manager® and ProduXion Manager® from Aries Systems Corporation

accepted. Please make sure it is accurate. View published research articles from *PLOS Medicine* for specific examples.<br><br>**NO authors have competing interests**<br><br>Enter: *The authors have declared that no competing interests exist.*<br><br>**Authors with competing interests**<br>Enter competing interest details beginning with this statement:<br><br>*I have read the journal's policy and the authors of this manuscript have the following competing interests: [insert competing interests here]*<br><br>* typeset<br><br>This statement is required for submission and **will appear in the published article** if the submission is accepted. Please make sure it is accurate and that any funding sources listed in your Funding Information later in the submission form are also declared in your Financial Disclosure statement.	
**Ethics Statement**<br><br>You must include an Ethics Statement in the Methods section of your manuscript if your study involved:<br>• Human participants<br>• Human specimens or tissue<br>• Vertebrate animals or cephalopods<br>• Vertebrate embryos or tissues<br>• Field research<br><br>**Not including a statement when required will delay the review process.**	I confirm that I have read and understood PLOS' requirements for an Ethics Statement.

General guidance is provided below. Please consult the guidelines on human subjects research and animal research for detailed instructions.

Format for specific study types

Human Subject Research (involving human participants and/or tissue)

- Give the name of the institutional review board or ethics committee that approved the study
- Include the approval number and/or a statement indicating approval of this research
- Indicate the form of consent obtained (written/oral) or the reason that consent was not obtained (e.g. the data were analyzed anonymously)

Animal Research (involving vertebrate animals, embryos or tissues)

- Provide the name of the Institutional Animal Care and Use Committee (IACUC) or other relevant ethics board that reviewed the study protocol, and indicate whether they approved this research or granted a formal waiver of ethical approval
- Include an approval number if one was obtained
- If the study involved *non-human primates*, add *additional details* about animal welfare and steps taken to ameliorate suffering

Field Research

Include the following details if this study involves the collection of plant, animal, or other materials from a natural setting:

- Field permit number
- Name of the institution or relevant body that granted permission

Please check the box to confirm your understanding of this policy, then read and agree to one of the statements.

Powered by Editorial Manager® and ProduXion Manager® from Aries Systems Corporation

Please respond:<br>as follow-up to "**Ethics Statement**<br><br>You must include an Ethics Statement in the Methods section of your manuscript if your study involved:<br>• Human participants<br>• Human specimens or tissue<br>• Vertebrate animals or cephalopods<br>• Vertebrate embryos or tissues<br>• Field research<br><br>**Not including a statement when required will delay the review process.**<br><br>General guidance is provided below. Please consult the guidelines on human subjects research and animal research for detailed instructions.	My study requires an ethics statement. I confirm I have included my statement in the Methods section of my uploaded manuscript file.

**Format for specific study types**<br><br>**Human Subject Research (involving human participants and/or tissue)**<br>• Give the name of the institutional review board or ethics committee that approved the study<br>• Include the approval number and/or a statement indicating approval of this research<br>• Indicate the form of consent obtained (written/oral) or the reason that consent was not obtained (e.g. the data were analyzed anonymously)<br><br>**Animal Research (involving vertebrate animals, embryos or tissues)**<br>• Provide the name of the Institutional Animal Care and Use Committee (IACUC) or other relevant ethics board that reviewed the study protocol, and indicate whether they approved this research or granted a formal waiver of ethical approval<br>• Include an approval number if one was obtained<br>• If the study involved *non-human primates*, add *additional details* about animal welfare and steps taken to ameliorate suffering<br><br>**Field Research**<br><br>Include the following details if this study involves the collection of plant, animal, or other materials from a natural setting:<br>• Field permit number<br>• Name of the institution or relevant body that granted permission<br><br>Please check the box to confirm your understanding of this policy, then read and agree to one of the statements. "	
**Data Availability**<br><br>Authors are required to make all data underlying the findings described fully available, without restriction, and from the time of publication. PLOS allows rare exceptions to address legal and ethical concerns. See the PLOS Data Policy and	No - some restrictions will apply

FAQ for detailed information.<br><br>A Data Availability Statement describing where the data can be found is required at submission. Your answers to this question constitute the Data Availability Statement and **will be published in the article**, if accepted.<br><br>**Important:** Stating 'data available on request from the author' is not sufficient. If your data are only available upon request, select 'No' for the first question and explain your exceptional situation in the text box.<br><br>Do the authors confirm that all data underlying the findings described in their manuscript are fully available without restriction?	
**Describe where the data may be found in full sentences. If you are copying our sample text, replace any instances of XXX with the appropriate details.**<br><br>• If the data are **held or will be held in a public repository**, include URLs, accession numbers or DOIs. If this information will only be available after acceptance, indicate this by ticking the box below. For example: *All XXX files are available from the XXX database (accession number(s) XXX, XXX.*).<br>• If the data are all contained **within the manuscript and/or Supporting Information files**, enter the following: *All relevant data are within the manuscript and its Supporting Information files.*<br>• If neither of these applies but you are able to provide **details of access elsewhere**, with or without limitations, please do so. For example:<br><br>*Data cannot be shared publicly because of [XXX]. Data are available from the XXX Institutional Data Access / Ethics Committee (contact via XXX) for*	The data may be shared on reasonable request but will need institutional approvals and agreements. Please contact the corresponding author to access the data.

researchers who meet the criteria for access to confidential data.<br><br>*The data underlying the results presented in the study are available from (include the name of the third party and contact information or URL).*<br>• This text is appropriate if the data are owned by a third party and authors do not have permission to share the data.<br><br>* typeset	
Additional data availability information:	Tick here if your circumstances are not covered by the questions above and you need the journal's help to make your data available.

Main manuscript

Click here to access/download;Manuscript;Plos Medicine ICE manuscript_v3.docx

Identifying novel prodromal symptoms of eclampsia: A multi-national, case-cohort study of prospectively collected data

Short title: Novel prodromal symptoms of eclampsia

Authors:

Roxanne Hastie[1,2], PhD, Farhatulain Ahmed[3], MD, Parinaz Mehdipour[1,2,4], PhD, Bernard Yan[5,6], DMedSc, Susan Walker[1,2], MD, Jacqui Visser[8], Anam Bashir[3], Lyle Gurrin[7], PhD, Anthea Lindquist[1,2], DPhil, Jess Atkinson[1,2], BBiomed (Hons), Catherine Cluver*[1,8], PhD, Lina Bergman[8,9]*, PhD, Stephen Tong[1,2]*, PhD.

[1] Mercy Perinatal, Mercy Hospital for Women, Heidelberg, Victoria, Australia

[2] Department of Obstetrics, Gynaecology and Newborn Health, University of Melbourne, Victoria, Australia

[3] Fatima Memorial Hospital, Lahore, Punjab, Pakistan

[4] Biostatistics Unit, Faculty of Health, Deakin University, Burwood, Victoria, Australia

[5] Department of Neurology, Royal Melbourne Hospital

[6] Department of Medicine, University of Melbourne

[7] Melbourne School of Population and Global Health, University of Melbourne

[8] Stellenbosch University, Cape Town, South Africa

[9] University of Gothenburg, Gothenburg, Sweden

*Authors contributed equally

Corresponding Author: Professor Stephen Tong. University of Melbourne, Level 4, Mercy Hospital for Women, 163 Studley Rd., Heidelberg 3084, Victoria, Australia. +613 84584444

stong@unimelb.edu.au

ABSTRACT

Background: Magnesium sulphate halves the risk of eclampsia. There is no consensus on who to give magnesium sulphate prophylaxis because clinical tools are poor at identifying those at risk. Known prodromal symptoms such as headache, visual disturbance or epigastric pain have modest associations with eclampsia. We set out to identify new prodromal symptoms of eclampsia.

Methods and findings: This case-control study prospectively recruited participants in South Africa and Pakistan who had eclampsia, preeclampsia or normotensive pregnancies. We asked whether they experienced 20 neurological symptoms, within 7 days of the seizure for those who had eclampsia. The primary analysis was the likelihood of symptoms occurring before eclampsia, compared to being present with preeclampsia.

341 participants were recruited with eclampsia, 1355 with preeclampsia and 389 with normotensive pregnancies. When comparing symptoms among those who had eclampsia versus preeclampsia, the odds ratios (OR) were 2.56 (95%CI 1.81-3.62) for headache, 5.73 (95%CI 4.44, 7.39) for visual disturbances and 2.25 (95%CI 1.76, 2.89) for epigastric pain. We identified ten symptoms with odds ratios over 10 for eclampsia. Odds ratios were 42.03 (95%CI 23.66, 74.68) for twitching/jerking limbs (30.5% eclampsia vs 1% preeclampsia), 33.12 (95%CI 19.46, 54.37) for affected hearing (21.1% vs 0.7%), 33.60 (95%CI 21.39, 52.78) for altered mind state (38.7% vs 1.8%); 33.12 for impaired speech, 23.71 for feelings of doom, 26.59 for severe vertigo, 20.52 for confusion, 18.16 for jitters, 15.81 for difficulty concentrating and 10.49 for weakness/paralysis. These symptoms were rare among normotensive pregnancies. Only 2.4% of women with eclampsia did not experience any prodromal symptoms.

Conclusions: Ten prodromal symptoms exhibit far stronger associations with eclampsia than headache, visual disturbances or epigastric pain. Eclampsia is uncommon without any prodromal symptoms. It may be useful to screen these symptoms among women with preeclampsia as part of

clinical history taking to guide management. They could help direct magnesium sulphate prophylaxis at those with a higher risk of eclampsia.

Introduction

Eclampsia is a tonic-clonic convulsion arising from neurological injury caused by severe preeclampsia[1]. With a prevalence of 1-2% among women with preeclampsia[2], it can cause maternal death[3], serious morbidity such as intracerebral haemorrhage[4] or persisting neurological deficits[5]. While more frequent in low- and middle-income countries (50-151/10,000 births), it also occurs in higher resource settings (1-10/10,000 births)[1,6].

Headache, visual changes and epigastric pain are prodromal symptoms ubiquitously used as part of clinical history among women with preeclampsia to infer eclampsia risk. The presence of these symptoms' means preeclampsia should be considered 'preeclampsia with severe features', according to the American College of Obstetricians and Gynaecologists Guidelines[7]. Furthermore, those who develop these symptom may be offered magnesium sulphate because this medication reduces the risk of eclampsia by 58%[2] and may reduce maternal death[2,8]. However, a meta-analysis concluded these symptoms have modest likelihood ratios in predicting eclampsia[9]. The likelihood ratios for eclampsia risk were 2.25 (95% CI 0.61-1.03) for headache; 5.81 (1.74-19.42) for visual disturbance and 3.4 (1.02-11.31) for epigastric pain. Furthermore, small studies have reported that 20%[10] or 41%[11] of eclamptic seizures occur without these prodromal symptoms.

We set out to find new prodromal symptoms of eclampsia that may have stronger associations than these existing symptoms that are widely used in the clinic. To do this we undertook a large prospective study running simultaneously in Pakistan and South Africa to characterise the association between 20 neurological symptoms and their occurrence before the onset of eclampsia. We considered the known prodromal symptoms of headache, visual changes and epigastric pain. But we also screened for symptoms not previously linked with this condition, but rather, occur with other neurological maladies (we screened for symptoms provided by B.Y., a neurologist). We

compared their likelihood of being present before an eclamptic seizure versus being present among women with preeclampsia who did not experience eclampsia. We also determined how often they occurred in normotensive pregnancies.

Methods

Study design, setting and population

This multi-site, case-control study prospectively recruited women across three tertiary referral centres: Fatima Memorial Hospital and Jinnah Hospital in Lahore, Pakistan; and Tygerberg Hospital in Cape Town, South Africa. Between 2018 – 2023, we recruited pregnant or postpartum women who experienced eclampsia within the last 7 days, women with preeclampsia who had not experienced eclampsia in the current pregnancy and women with normotensive pregnancies. Eclampsia was defined as the new onset of tonic-clonic seizures among pregnant or newly postpartum women with preeclampsia. Preeclampsia was defined according to the International Society for the Study of Hypertension in Pregnancy[12] as hypertension (>140 mmHg systolic blood pressure or > 90 mmHg diastolic blood pressure) after the 20th week of gestation and the presence of one or more of the following new onset conditions: proteinuria, renal insufficiency (creatinine >90 umol/L), liver involvement (elevated transaminases and/or severe right upper quadrant or epigastric pain), neurological complications, haematological complications (thrombocytopenia, disseminated intravascular coagulation, haemolysis) or fetal growth restriction. In the South African cohort, proteinuria had to be present for a diagnosis of preeclampsia. Normotensive pregnant or postpartum women were those without hypertension or eclampsia. We did not include women with multifetal pregnancies, those with seizures attributed to a diagnosis other than eclampsia (such as central nervous system infections, a history of seizures or epilepsy) and women unable to provide informed consent. To avoid crossover, we excluded women with preeclampsia who were enrolled and undertook the survey but subsequently developed eclampsia, and we excluded those who were enrolled as normotensive when they undertook the survey but who later developed a hypertensive disorder or eclampsia.

All the authors contributed to the study design, vouch for the completeness and accuracy of the data, and have agreed to submit the manuscript for publication. The senior and first authors wrote all drafts of the manuscript. The protocol was shared with an Egyptian team who are undertaking an independent investigation of these symptoms.

Procedures

Women presenting to each site with eclampsia were identified by the research team. Women with preeclampsia and normotensive pregnant women were recruited among those seeking maternity services. When clinically stable, women were approached about the study and invited to participate. Those willing and able to provide informed consent were enrolled into the study.

At enrolment, all participants were asked a series of standardised structured interview questions (translated to the local language at each site) about symptoms they experienced prior to seizure onset for those with eclampsia and prior to enrolment for women with preeclampsia and normotensive pregnancies. The symptoms investigated were those previously associated with eclampsia: headache, epigastric pain, visual disturbances and nausea and vomiting. Other symptoms investigated were those typically associated with neurological disorders outside of pregnancy including memory loss, difficulty concentrating, confusion, mood changes, twitching or jerking of limbs, hearing or speech impairment, muscle weakness or paralysis. The complete list of symptoms screened in our survey are listed in Supplementary Table 1.

Maternal demographics and clinical characteristics were also obtained from maternal interview and review of medical records. Participants were followed from enrolment until discharge from hospital

with birthing and maternal and neonatal outcome data obtained. All data was captured using the secure online database Research Electronic Data Capture (REDCap).

Statistical analysis

Statistics summarising the distribution of clinical characteristics were prepared for women with eclampsia, preeclampsia and those with normotensive pregnancies.

As per our a priori protocol (Supplemental File 2), the primary analysis compared the prevalence of prodromal symptoms between women with eclampsia to those with preeclampsia. The magnitude of the association between each prodromal symptom and the likelihood of eclampsia was estimated as an odds ratio (with 95% confidence intervals) from a univariable logistic regression model.

A similar analysis was performed comparing the prevalence of prodromal symptoms between women with eclampsia and normotensive women. The frequency and number of symptoms were also compared qualitatively between women with eclampsia and i) preeclampsia and ii) normotensive women without formal statistical testing. Analyses were performed using Stata (version 18.0) and R (version 4.4.3).

Role of the funding source

The funders had no role in the design and conduct of the study; collection, management, analysis, and interpretation of the data; preparation, review, or approval of the manuscript; and decision to submit the manuscript for publication.

Results

Between 2018 to 2023 we recruited 2,142 participants. 341 had eclampsia, 1355 had preeclampsia and 389 had normotensive pregnancies. Among those with preeclampsia, 23 developed eclampsia after recruitment and were excluded from the primary analysis. Similarly, among normotensive women 22 developed hypertension or preeclampsia and were also excluded. Additionally, 12 women with eclampsia were recruited more than 7 days after experiencing an eclamptic seizure and were excluded from analysis (Figure 1). Of those recruited during pregnancy, gestational ages at recruitment were similar among all cohorts. Compared to those who had preeclampsia or normotensive pregnancies those who had eclampsia were younger, had a lower body mass index, were more often nulliparous or booked later for antenatal care, or did not receive any antenatal care (Table 1 and Supplementary Table 2).

1453 (69.7%) were recruited from Cape Town, South Africa and 632 (30.3%) from Lahore, Pakistan (baseline information by country shown on Supplementary Table 3). Participants from the two countries were similar in age, gestation at recruitment and other baseline characteristics but there were differences in ethnicity (In South Africa, 64.0% Black, 35.3% Bi-racial, 0.6% Caucasian whereas in Pakistan, 100% were South Asian) and the numbers that did not receive antenatal care (6.6% in South Africa versus 43.4% in Pakistan).

Our primary comparison was the likelihood of symptoms being present before an eclamptic seizure versus being present among women with preeclampsia (Table 2). The odds ratio (OR) for a headache preceding an eclamptic seizure was 2.56 (95% CI 1.81, 3.62; 88.0% eclampsia vs 74.1% preeclampsia) – Table 2. There was a stepwise increase in the odds ratios with increasing headache severity (supplementary table 4). A sudden headache was associated with a higher odds ratio (7.86 (95% CI 5.21-11.88) compared to a headache of gradual onset (OR 1.79 (95% 1.25-2.56)).

The odds ratios of visual disturbances preceding eclampsia was 5.73 (95% CI 4.44-7.39; 65.1% eclampsia vs 24.6% preeclampsia) and most often occurred within 24 hours of an eclamptic seizure (Supplementary table 4). The odds ratio for epigastric pain was 2.25 (95%CI 1.76-2.89; 41.9% eclampsia vs 24.3% preeclampsia). There was modest increase in the odds ratio with increasing severity of pain (Supplementary Table 4). Headache, visual changes and epigastric pain were uncommon among normotensive pregnancies: headache 3.3%, visual disturbance 0.5% and epigastric pain 1.0% (Supplementary Table 5).

We identified ten prodromal symptoms that were present preceding eclampsia with odds ratios over 10, compared with being present with preeclampsia (Table 2). Odds ratios over 30 were: 42.03 for twitching or jerking (95% CI 23.66, 74.68; 30.5% present prior to eclampsia versus 1.0% present with preeclampsia); 36.00 for affected hearing (95% CI 18.34, 70.65; 21.1% eclampsia versus 0.7% preeclampsia), 33.60 for affected mind state (95% CI 21.39, 52.78; 38.7% eclampsia versus 1.8% preeclampsia) and 33.12 for affected speech (95% CI 19.46, 54.37; 29.6% eclampsia versus 1.2% preeclampsia). Odds ratios over 20 were: 26.59 for severe vertigo (95% CI 7.82, 90.41; 5.6% eclampsia versus 0.2% preeclampsia), 23.71 for feelings of doom (95% CI 16.49, 34.10; 45.4% eclampsia versus 3.4% preeclampsia) and 20.52 for confusion (95% CI 14.22, 29.63; 41.3% eclampsia versus 3.3% preeclampsia). Odds ratios between 10-20 were for: 18.16 (95% CI 10.76, 30.66) for jitters/nervous shaking, 15.18 (95% CI 11.34, 20.33) for difficulty concentrating and 10.49 (95% CI 6.76, 16.27) for weakness or paralysis.

When the data were split according to whether they were collected in Pakistan or South Africa, all ten symptoms retained odds ratios above 10 at both sites, except weakness or paralysis (odds ratio 12.53 in Pakistan and 7.07 in South Africa; Figure 2). Conversely, the known prodromal symptoms of headache, visual changes and epigastric pain all had odds ratios less than 10 at both sites.

These novel prodromal symptoms rarely occurred among those who had normotensive pregnancies. Among all those with normotensive pregnancies, twitching and jerking, or confusion occurred once, and the remaining top prodromal symptoms were not reported at all (Supplementary Table 5). The only odds ratios estimable, for twitching and jerking among those who had eclampsia compared to those with normotensive pregnancies, was 170.26 (95% CI 23.60, 1228.21; 30.5% eclampsia versus 0.3% normotensive).

We identified other novel prodromal symptoms more likely to be present with eclampsia compared with preeclampsia, but with lower odds ratios. They were severe dizziness, feeling anxious, changes in mood, nausea and vomiting, tightness in the chest, edema or shortness of breath, and focal neurological deficits not including vertigo (Supplementary Table 4). They were uncommon among women with normotensive pregnancies (Supplementary Table 5).

Eclampsia was uncommon without prodromal symptoms. Only 2.4% (8/341) of those who had eclampsia did not report any of the 20 prodromal symptoms we screened for in this study (Table 3). When confining our analysis to 13 prodromal symptoms (headache, visual changes, epigastric pain, plus the ten symptoms with odds ratios >10), eclampsia occurred in the absence of these symptoms in only 6.1% of women (21/341; Table 3). Eclampsia was more often associated with the presence of many co-existing prodromal symptoms, compared to preeclampsia (Table 3). Four or more prodromal symptoms were present in 60.5% of those who had eclampsia but only 6.2% who had preeclampsia. Conversely, 2 symptoms or less occurred in 27.8% of those who had eclampsia but 85.1% among those who had preeclampsia.

Discussion:

It is uncommon to uncover new symptoms for any condition, especially for afflictions that have been known about for centuries[20]. In this prospective study done in two regions of the globe, we identified ten new prodromal symptoms strongly associated with eclampsia, with odds ratios over 10 when compared to women with preeclampsia. These new symptoms were more strongly associated with eclampsia than headache, visual changes or epigastric pain, prodromal symptoms widely used in the clinic as part of daily history taking from women with preeclampsia, or hypertension during pregnancy. The presence of an increasing number of symptoms made eclampsia more likely. Conversely, the occurrence of eclampsia without prodromal symptoms was infrequent.

Incorporating these ten symptoms as part of history taking may be useful as part of clinical management of women with preeclampsia or hypertension during pregnancy. Their presence may suggest preeclampsia with neurological involvement. Importantly, their presence may mean there is an elevated risk of eclampsia.

While this was not intended to be a diagnostic prediction study, the symptoms we have identified may be useful to direct who to give magnesium sulphate, in the same way existing symptoms (with far lower odds ratios) are currently used[13-15]. For instance, the sudden onset of twitching or arm jerking (OR 42.03), affected hearing (OR 36.0) and confusion (OR 20.52) in a woman with preeclampsia might raise concerns she is at elevated risk of eclampsia where the commencement of magnesium sulphate is reasonable. In contrast, those with preeclampsia but who are asymptomatic appear to be at very low risk.

Currently, there is no consensus which pregnancies complicated by preeclampsia should be offered magnesium sulphate. Without reliable clinical indicators to predict eclampsia, recommendations

from international guidelines vary widely regarding who to offer magnesium sulphate. Some recommend magnesium sulphate if prodromal symptoms (specifically headache, visual changes or epigastric pain) or other features are present, such as severe hypertension[13-15] or if there is progressive deterioration in laboratory blood tests[14,15]. Others do not base their recommendations on the presence of symptoms: The American College of Obstetricians and Gynecologists (ACOG) guidelines recommend magnesium sulphate is offered to women with preeclampsia and severe features[16] (this would include headache or visual changes, as these are considered severe features) while The World Health Organisation recommends magnesium sulphate is given to all women with preeclampsia, even mild disease[17].

Classic prodromal symptoms of headache, visual disturbances and epigastric pain were only modestly associated with eclampsia. Their generally high prevalence in preeclamptic pregnancies may be why they performed modestly. In contrast, the absolute incidence of the ten new symptoms we report were low with preeclampsia but high with eclampsia.

The absence of any prodromal symptoms may mean the eclampsia risk is very low. Hence, for those with preeclampsia without neurological symptoms, it may be reasonable to withhold magnesium sulphate, especially in high income settings where the background prevalence is low[1,6]. Such a selective approach to offering magnesium sulphate based on the presence of symptoms may reduce numbers given the drug and this could reduce healthcare costs.

Our study had several limitations. While we recruited participants prospectively, we asked about prodromal symptoms after the seizure happened. However, a prospective screening study to find new symptoms would have been highly challenging. Eclampsia is rare, even in low-middle income settings meaning extremely large numbers would need to be screened. Another limitation is that clinicians may have commenced magnesium sulphate if the classic prodromal symptoms of

headache and visual disturbances were present, but not for the novel symptoms we have identified. This may have introduced interventional bias and reduced the apparent strength of association with eclampsia for these known prodromal symptoms.

Another potential limitation is the risk of recall bias. Those affected by eclampsia will have had a significant neurological episode meaning they might have recalled symptoms differently from those with preeclampsia or were normotensive. However, the relative differences in odds ratios between symptoms within groups may still be valid for two reasons. First, the entire symptom list was screened within groups meaning all symptoms would be equivalently prone to recall bias. Second, the novel prodromal symptoms had consistently higher odds ratios compared with known symptoms when the data was split according to country collected (Figure 2), and this provides validity.

Strengths of our study were that we recruited participants within seven days of an eclamptic seizure or while they still had preeclampsia, that a research midwife interviewed the participants, and we used the same questionnaire for both countries. We were agnostic as to which novel symptom could be associated with eclampsia, which minimised bias.

The new symptoms we identified do not localise to a specific region of the brain such as the posterior compartment, suggesting the pathophysiology of eclampsia is diffuse. This is in keeping with eclampsia being a maternal vascular lesion that impairs vessel autoregulation and can affect any region of the brain[1,18,19]. Also, the prevalence of the novel symptoms we described were high in the eclamptic cohort, less in the preeclampsia cohort but notably, hardly occurred in normotensive pregnancies. This hints at a biological gradient some degree of neuronal pathology exists in cases of preeclampsia where there are neurological symptoms, even in the absence of seizures.

In summary, in this two-country prospective study, we identified new prodromal symptoms strongly associated with eclampsia. Eclampsia was uncommon in the absence of any prodromal symptoms. These new symptoms we have uncovered may be useful as part of history taking to more accurately assess likely eclampsia risk. It could be considered as part of the whole clinical picture to judge whether delivery is indicated in cases of preterm preeclampsia. Finally, these symptoms may be useful in deciding whether magnesium sulphate is required or could be safely withheld.

Acknowledgements: We thank all the women that participated in this study, the staff at Fatima Memorial Hospital and Jinnah Hospital in Lahore, Pakistan; and Tygerberg Hospital in Cape Town, South Africa. This study supported via the RANZCOG Women's Health Foundation. RH, ST and were all supported by the National Health and Medical Research Council. LB is a Wallenberg Scholar, supported by the Wallenberg Center for Molecular and Translational Medicine (WCMTM). CC receives salary support from the Mercy Health Foundation. The funders had no role in the design and conduct of the study; collection, management, analysis, and interpretation of the data; preparation, review, or approval of the manuscript; and decision to submit the manuscript for publication.

Declaration of interests: Drs Tong, Cluver and Walker provide consultancy services for Diamedica Therapeutics. Drs Cluver and Tong provide consultancy services for Avilar Therapeutics.

Contributions statement. ST, CC and LB contributed equally to this report. ST and RH conceptualised the trial. BY was the neurologist who helped generate the survey (with ST, RH) by proposing novel neurological symptoms to screen. FA and AB led recruitment and data collection in Pakistan; while CC, LB, JV led recruitment and data collection in Cape Town. SW provided senior intellectual input throughout the study and assisted with study oversight. PM, RH led data analysis with senior

intellectual input from LG. ST and RH wrote the first draft, and JA assisted with re-drafting of the report. All authors provided intellectual input in data interpretation, drafting of the manuscript and approved the final version. The corresponding author attests that all listed authors meet authorship criteria and that no others meeting the criteria have been omitted.

Data access statement: RH and PM accessed and verified the underlying data reported in the whole manuscript. LB, CC and JV accessed and verified the data from Cape Town and FA and AB accessed and verified the data from Pakistan. All authors had access to the data and accept responsibility to submit for publication.

References:

1. Fishel Bartal M, Sibai BM. Eclampsia in the 21st century. *Am J Obstet Gynecol* 2022; **226**(2S): S1237-S53.
2. Altman D, Carroli G, Duley L, et al. Do women with pre-eclampsia, and their babies, benefit from magnesium sulphate? The Magpie Trial: a randomised placebo-controlled trial. *Lancet* 2002; **359**(9321): 1877-90.
3. Abalos E, Cuesta C, Carroli G, et al. Pre-eclampsia, eclampsia and adverse maternal and perinatal outcomes: a secondary analysis of the World Health Organization Multicountry Survey on Maternal and Newborn Health. *BJOG* 2014; **121 Suppl 1**: 14-24.
4. Di X, Mai H, Zheng Z, Guo K, Morse AN, Liu H. Neuroimaging findings in women who develop neurologic symptoms in severe preeclampsia with or without eclampsia. *Hypertens Res* 2018; **41**(8): 598-604.
5. Andersgaard AB, Herbst A, Johansen M, Borgstrom A, Bille AG, Oian P. Follow-up interviews after eclampsia. *Gynecol Obstet Invest* 2009; **67**(1): 49-52.
6. Berhan Y, Berhan A. Should magnesium sulfate be administered to women with mild pre-eclampsia? A systematic review of published reports on eclampsia. *J Obstet Gynaecol Res* 2015; **41**(6): 831-42.
7. Pregnancy ATfoHi. Hypertension in Pregnancy. Washington: ACOG; 2013.
8. McDonald SD, Lutsiv O, Dzaja N, Duley L. A systematic review of maternal and infant outcomes following magnesium sulfate for pre-eclampsia/eclampsia in real-world use. *Int J Gynaecol Obstet* 2012; **118**(2): 90-6.
9. Hastie R, Brownfoot FC, Cluver CA, et al. Predictive Value of the Signs and Symptoms Preceding Eclampsia: A Systematic Review. *Obstet Gynecol* 2019; **134**(4): 677-84.
10. Cooray SD, Edmonds SM, Tong S, Samarasekera SP, Whitehead CL. Characterization of symptoms immediately preceding eclampsia. *Obstet Gynecol* 2011; **118**(5): 995-9.
11. Douglas KA, Redman CW. Eclampsia in the United Kingdom. *BMJ* 1994; **309**(6966): 1395-400.
12. Brown MA, Magee LA, Kenny LC, et al. Hypertensive Disorders of Pregnancy: ISSHP Classification, Diagnosis, and Management Recommendations for International Practice. *Hypertension* 2018; **72**(1): 24-43.
13. SOMANZ. Society of Obstetric medicine Australia and New Zealand Hypertension in Pregnancy Guideline, Sydney; 20232023. (accessed.
14. NICE. NICE Guideline [NG133] Hypertension in pregnancy: diagnosis and management 2019. https://www.nice.org.uk/guidance/ng133 (accessed.
15. Magee LA, Brown MA, Hall DR, et al. The 2021 International Society for the Study of Hypertension in Pregnancy classification, diagnosis & management recommendations for international practice. *Pregnancy Hypertens* 2022; **27**: 148-69.
16. Gestational Hypertension and Preeclampsia: ACOG Practice Bulletin, Number 222. *Obstet Gynecol* 2020; **135**(6): e237-e60.
17. Organisation WH. WHO recommendations for prevention and treatment of pre-eclampsia and eclampsia. In: Organisation WH, editor.; 2011.
18. Bergman L, Cluver C, Carlberg N, et al. Cerebral perfusion pressure and autoregulation in eclampsia-a case control study. *Am J Obstet Gynecol* 2021; **225**(2): 185 e1- e9.
19. Bergman L, Hannsberger D, Schell S, et al. Cerebral infarcts, edema, hypoperfusion, and vasospasm in preeclampsia and eclampsia. *Am J Obstet Gynecol* 2024.
20. Chesley LC. History and epidemiology of preeclampsia-eclampsia. *Clin Obstet Gynecol* 1984; **27**(4): 801-20.

Characteristic	Eclampsia (N=341)	Preeclampsia (N=1,355)	Normotensive (N=389)
**Maternal Characteristics**			
**Maternal age, mean (SD)**	25.0 (6.1)	28.4 (6.2)	28.6 (6.1)
Missing	0 (0%)	0 (0%)	2 (0.5%)
**Race n (%)**			
Black	109 (32.0)	625 (46.1)	196 (50.4%)
Biracial	48 (14.1)	316 (23.3)	149 (38.3%)
Caucasian	-	5 (0.4)	3 (0.8%)
Indian	-	1 (0.07)	0 (0%)
South Asian	184 (54.0)	408 (30.1)	41 (10.5%)
**Country n (%)**			
Pakistan	183 (53.7)	408 (30.1%)	41 (10.5%)
South Africa	158 (46.3)	947 (69.9%)	348 (89.5%)
**Body mass index, mean (SD)**	27.0 (5.7)	29.6 (7.3)	28.1 (7.5)
Missing	27 (7.9%)	75 (5.5%)	24 (6.2%)
**Gestation at inclusion (week; median (IQR))***	34.8 (31.0, 37.1)	34.9 (31.7, 37.4)	34.7 (29.7, 38.6)
Missing	32 (9.4%)	73 (5.4%)	10 (2.6%)
**Nulliparous n(%)**	211 (61.9%)	599 (44.2%)	112 (28.8%)
Missing	0 (0%)	1 (0.07%)	0 (0%)
**Antenatal care n(%)**			
None	120 (35.2%)	234 (17.3%)	16 (4.1%)
After 20 weeks	96 (28.1%)	358 (26.4%)	111 (28.5%)
Before 20 weeks	122 (35.8%)	753 (55.6%)	258 (66.3%)
Missing	3 (0.9%)	10 (0.7%)	4 (1.0%)
**Highest systolic blood pressure (median (IQR))**	170 (160, 185.5)	165 (153, 180)	120 (110.0, 126.0)
**Highest diastolic blood pressure (median (IQR))**	110 (100, 120)	101 (95, 110)	70 (62, 76)
Missing	1 (%)	4 (%)	19 (%)
**Anti-hypertensive treatments at booking (%)**	33 (9.7%)	140 (10.3%)	0 (0%)
**Tobacco use (%)**	17 (5.0%)	88 (6.5%)	51 (13.1%)
**Neonatal Characteristics**			
**Stillborn (%)**	57 (16.7%)	113 (8.3%)	10 (2.6%)
Missing	2 (0.6)	92 (6.8%)	8 (2.1%)

**Sex (%)**			
Female	162 (47.5)	650 (48.0)	209 (53.6%)
Missing	6 (1.8%)	127 (9.4%)	8 (2.1%)
**Birthweight (median (IQR))**	2152.5 (1500, 2800)	2080 (1370, 2730)	2900 (2170, 3275)
Missing	11 (3.2%)	128 (9.4%)	12 (3.1%)
**Gestational age at birth (median (IQR))**	35.3 (31.9, 37.7)	35.1 (32.0, 37.6)	38.1 (34.8, 39.3)
Missing	70 (20.5%)	134 (9.9%)	13 (3.3%)
**Mode of birth (%)^**			
Vaginal birth	87 (25.5%)	318 (23.5%)	214 (55.0%)
Cesarean section	249 (73.0%)	923 (68.1%)	168 (43.2%)
Missing	4 (1.2%)	114 (8.4%)	7 (1.8%)

Table 1: Participant characteristics among pregnancies complicated by preeclampsia, eclampsia or neither (normotensive pregnancies). *263 participants were recruited postpartum. ^1 case of antepartum maternal death where the fetus remained undelivered.

Symptom	Eclampsia (N=341)	Preeclampsia (N=1,355)	Odds Ratio (95% CI)
	**Known prodromal symptoms – N (%)**		
**Headache**	300 (88.0)	1004 (74.1)	2.56 (1.81 to 3.62)
**Visual disturbances**	222 (65.1)	333 (24.6)	5.73 (4.44 to 7.39)
**Epigastric pain**	143 (41.9)	329 (24.3)	2.25 (1.76 to 2.89)
	**New prodromal symptoms – N (%)**		
**Twitching or jerking of arms or legs**	105 (30.5)	14 (1.0)	42.03 (23.66 to 74.68)
**Hearing affected**	72 (21.1)	10 (0.7)	36.00 (18.34 to 70.65)
**Mind state affected**	132 (38.7)	25 (1.8)	33.60 (21.39 to 52.78)
**Speech affected**	101 (29.6)	17 (1.2)	33.12 (19.46 to 54.37)
**Severe vertigo**	19 (5.6)	3 (0.2)	26.59 (7.82 to 90.41)
**Feelings of impending doom or end of the world**	155 (45.4)	46 (3.4)	23.71 (16.49 to 34.10)
**Confusion**	141 (41.3)	45 (3.3)	20.52 (14.22 to 29.63)
**Jitters/nervousness/nervous shaking**	70 (20.5)	19 (1.4)	18.16 (10.76 to 30.66)
**Difficulty concentrating**	192 (56.3)	106 (7.8)	15.18 (11.34 to 20.33)
**Weakness or paralysis**	69 (20.2)	32 (2.4)	10.49 (6.76 to 16.27)

Table 2: Odds ratios for the presence of symptoms being present prior to the onset of eclampsia compared to being present among women with preeclampsia

Number of symptoms	Eclampsia (N=341)	Preeclampsia (N=1,355)	Normotensive (N=389)
**0**	**21 (6.1%)**	**278 (20.5%)**	**375 (95.4%)**
1	23 (6.7%)	554 (40.9%)	15 (3.9%)
2	51 (15.0%)	321 (23.7%)	2 (0.5%)
3	40 (11.7%)	118 (8.7%)	1 (0.3%)
4	34 (10.0%)	40 (3.0%)	0 (0%)
5	32 (9.4%)	16 (1.2%)	0 (0%)
6	27 (7.9%)	14 (1.0%)	0 (0%)
7	21 (6.2%)	4 (0.3%)	0 (0%)
8	29 (8.5%)	5 (0.4%)	0 (0%)
9	24 (7.0%)	3 (0.2%)	0 (0%)
10	18 (5.3%)	0 (0%)	0 (0%)
11	11 (3.2%)	2 (0.2%)	0 (0%)
12	4 (1.2)	0 (0%)	0 (0%)
13	6 (1.8)	0 (0%)	0 (0%)

Table 3: Frequency of 13 symptoms being present in the preeclampsia, eclampsia and normotensive cohorts.

Symptoms were headache, visual changes, epigastric pain, twitching/limb jerking, affected hearing, altered mind state, severe vertigo, impaired speech, feelings of doom, confusion, jitters, difficulty concentrating and weakness/paralysis.

Numbers and percentages shown.

Figure 1 Click here to access/download;Figure;Figure_1 v2 pptx.pptx

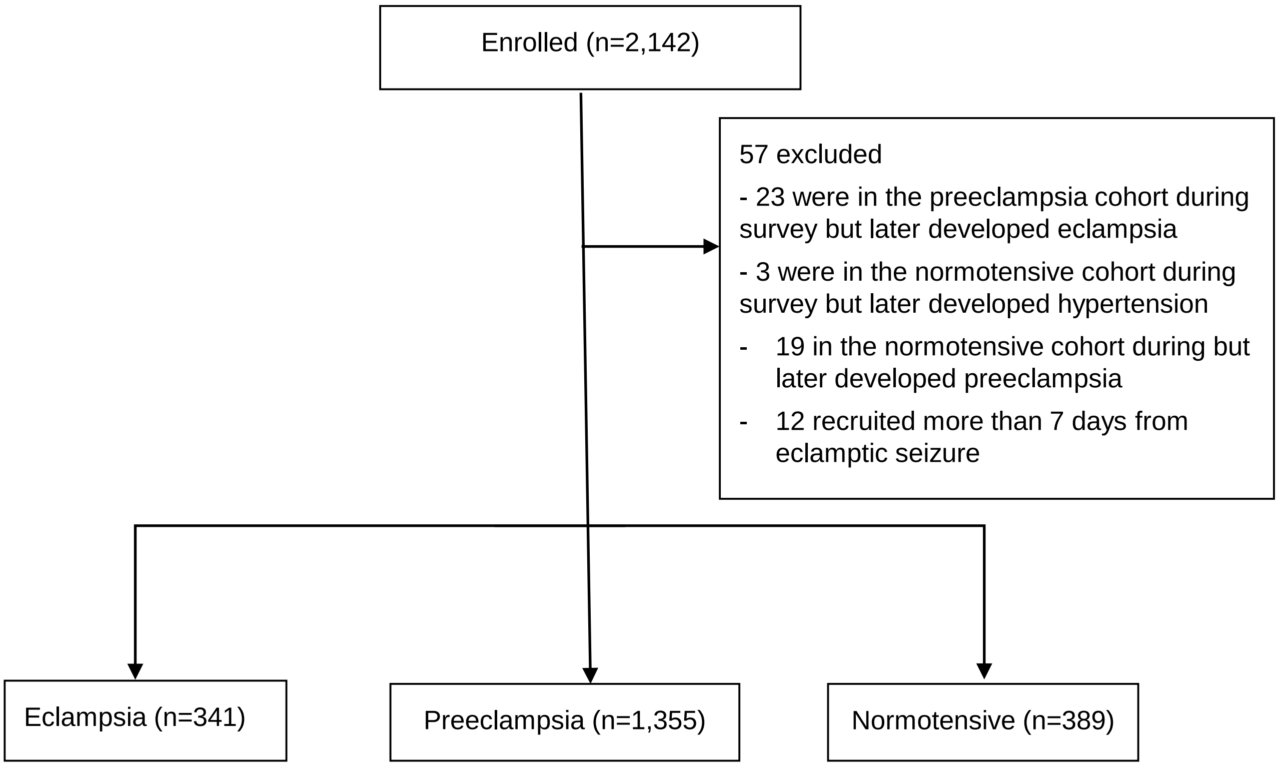

Figure 1: Recruitment of participants

Click here to access/download;Figure;Figure 2 .pptx

Symptom	Eclampsia N (%)	Odds Ratio (95% CI)
Epigastric pain	92 (50.3%)	1.83 (1.29 to 2.61)
Headache	163 (89.1%)	3.36 (2.01 to 5.59)
Visual disturbances	116 (63.4%)	5.95 (4.07 to 8.70)
Weakness or paralysis	41 (22.4%)	7.07 (3.85 to 13.00)
Confusion	79 (43.2%)	11.16 (6.81 to 18.28)
Difficulty concentrating	97 (53.0%)	11.66 (7.44 to 18.25)
Jitters/nervousness/shaking	51 (27.9%)	12.75 (6.60 to 24.64)
Speech affected	53 (29.0%)	16.23 (8.02 to 32.81)
Feelings of impending doom	74 (40.4%)	16.63 (9.31 to 29.72)
Severe vertigo	11 (6.0%)	26.03 (3.33 to 203.18)
Twitching/jerking of arms/legs	82 (44.8%)	29.3 (15.05 to 57.04)
Hearing affected	40 (21.9%)	37.76 (11.5 to 123.96)
Mind state affected	55 (30.1%)	43.4 (15.43 to 122.08)

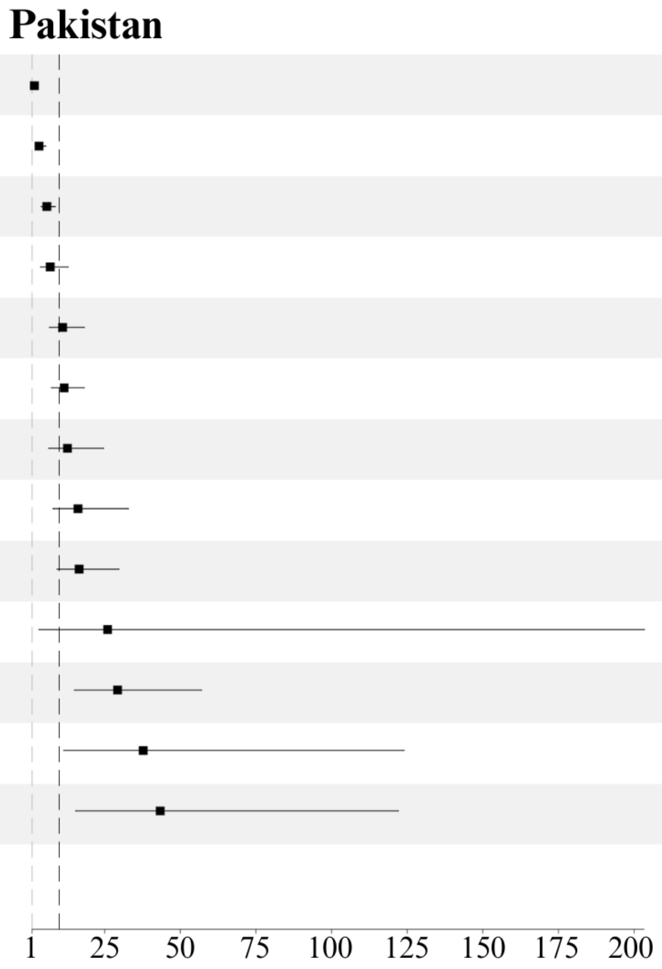

Symptom	Eclampsia N (%)	Odds Ratio (95% CI)
Epigastric pain	51 (32.3%)	1.98 (1.36, 2.86)
Headache	137 (86.7%)	2.12 (1.31, 3.43)
Visual disturbances	106 (67.1%)	5.97 (4.16, 8.58)
Weakness or paralysis	28 (17.7%)	12.53 (6.60, 23.79)
Jitters/nervousness/shaking	19 (12.0%)	18.36 (7.58, 44.46)
Difficulty concentrating	95 (60.1%)	18.89 (12.65, 28.21)
Severe vertigo	8 (5.1%)	25.20 (5.30, 119.81)
Confusion	62 (39.2%)	31.54 (18.10, 54.96)
Feelings of impending doom	81 (51.3%)	32.15 (19.91, 51.92)
Hearing affected	32 (20.3%)	34.10 (14.74, 78.89)
Mind state affected	77 (48.7%)	41.92 (24.59, 71.45)
Twitching/jerking of arms/legs	22 (13.9%)	50.90 (15.03, 172.34)
Speech affected	48 (30.4%)	58.60 (25.88, 132.67)

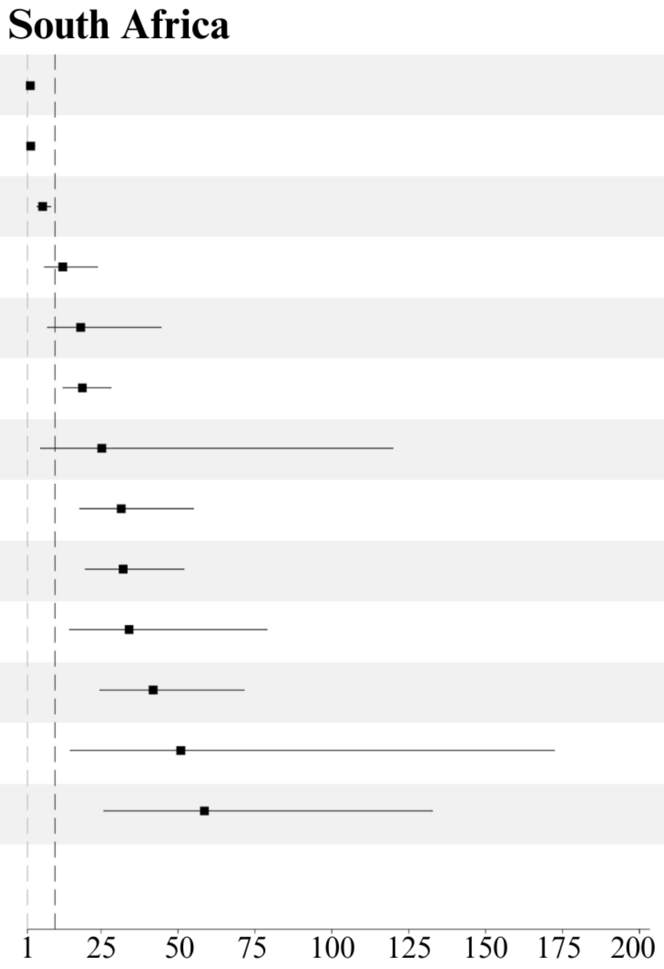

Figure 2: Odds ratios for the presence of symptoms prior to the onset of eclampsia compared to being present among women with preeclampsia, divided according to country of recruitment.

Click here to access/download
Supporting Information
Plos Medicine Supplementary tables_v2.docx

Click here to access/download
Supporting Information
STUDY PROTOCOL_ICE_V6.docx
