# Supplementary material for: Identifying novel prodromal symptoms of eclampsia: A two-country, case-control study
Source: PLoS Med. 2026 Apr 28;23(4):e1004994. doi: 10.1371/journal.pmed.1004994 (PMC13123989; doi:10.1371/journal.pmed.1004994)
Supplement: Attachment — Submitted filename: Comments_Alice Beardmore_Gray.docx [file pmed.1004994.s006.docx]

**Overall:**

This is a very interesting and impressive study which has the potential to alter clinical practice and guidelines. As the authors state, it is uncommon to uncover new symptoms for any condition, and this manuscript highlights the importance of continuing to question current practice and improve the body of established medical evidence – this is even more important in the often-neglected field of women’s health and this study contributes important, novel, findings to our understanding of a condition which remains a leading cause of maternal death globally. As an Obstetrician, I believe the findings offer real clinical utility; it would be great to understand more about how they could be taken further and incorporated into routine practice, as well as evaluated more widely and across different populations and settings. The identification of these 10 prodromal symptoms which may predict the onset of eclampsia has the potential to offer a simple, low-cost screening tool that could prompt earlier intervention and thus reduce maternal and perinatal mortality. There are a few points below which I feel could benefit from further detail, as well as two small typos which I have highlighted on the pdf.

Abstract:

1. Clear and informative

Introduction:

1. Also very clear and informative, clearly sets out the research question and provides helpful context, with concise definitions.

Methods:

1. It would be interesting to know why the two sites were selected, and if word count allowed, a brief description of the maternity units (e.g. number of deliveries, population served, world-bank income classification and health system structure e.g. publicly or privately funded, insurance-based etc.).
2. I’m also interested to know why a period of 7 days was chosen, though this seems logical given the clinical nature of pre-eclampsia and eclampsia and the need to balance contemporaneous reviews in a high-risk postnatal population whilst also allowing time for recovery
3. Was a sample size calculation performed?
4. I understand that the symptoms were discussed with a neurologist, however it would be interesting (especially for the non-neurologist) to understand why these particular 20 symptoms were chosen, and any evidence/literature justifying them.
5. Were there any issues in translating the interview questions, and can the team be sure that the symptoms were well-understood by participants particularly if speaking a different language and accounting for a different cultural context
6. How were the diagnoses of pre-eclampsia and eclampsia verified by the clinical teams?

Results:

1. 35.2% of women in the eclamptic cohort received no antenatal care; this lack of surveillance and potential earlier detection of pre-eclampsia (and eclampsia) may have contributed to the severity of their disease and presence of severe symptoms: it would be interesting to repeat this study in an even larger cohort of women across multiple different care settings, in order to establish whether screening for these prodromal symptoms is useful in all settings, or only those were there is a limited antenatal care and therefore later presentations to care with more severe disease.
2. It would also be interesting to establish whether symptom severity correlates with blood pressure and other markers of pre-eclampsia severity (biochemistry, ultrasound findings)
3. To have evaluated a cohort of 341 women who experienced eclampsia is hugely impressive – I hope that the editors appreciate that this is a relatively rare outcome, and thus to have a cohort of 341 women is excellent

Discussion:

1. The authors state that the ten symptoms identified as being strongly associated with eclampsia may be useful as part of clinical management of women with pre-eclampsia. I think it would be interesting to better understand how the authors envisage this could be incorporated into routine clinical practice. Do they propose a routine checklist at antenatal visits? Do they plan on assessing the feasibility of this? Have they undertaken any qualitative assessment of healthcare providers views on such a checklist and whether they feel it would be a helpful part of their assessment?
2. I also think it would be useful to evaluate these symptoms across a broader range of settings in order to establish the universality of such symptoms, and whether screening is more or less useful according to different settings with differing resources available.
3. A huge strength of this study, in my opinion, is the potential for a very simple, low-cost screening tool which could be implemented in low and middle income countries and potentially prompt earlier intervention. It would be useful to have more information about whether the authors feel there is sufficient evidence from their findings to support this, and whether there is scope to further evaluate this as a screening tool across other settings. I would be interested to explore this in Zambia and Sierra Leone.
