# Supplementary material for: Identifying novel prodromal symptoms of eclampsia: A two-country, case-control study
Source: PLoS Med. 2026 Apr 28;23(4):e1004994. doi: 10.1371/journal.pmed.1004994 (PMC13123989; doi:10.1371/journal.pmed.1004994)
Supplement: Attachment — Submitted filename: Second resubmission Rebuttal to Referee Comments.docx [file pmed.1004994.s010.docx]

Dear Suzanne De Bruijn, Associate Editor of PLOS Medicine,

We are pleased to respond to remaining questions posed by referees. We have also added an author summary to this draft.

Kind regards

Stephen Tong (corresponding author)

**Reviewer 1**

**Reviewer #1:**

**Thank you for addressing my comments. I have two more suggestions:**

**1. Please remove 'prospectively collected data' from the title - except for database studies, all case-control studies would be using prospectively collected data. This could confuse readers as 'prospective' is often used to describe prospective follow-up (which this study doesn't have).**

A: Done.

New title: Identifying novel prodromal symptoms of eclampsia: A multi-national, case-control study.

**2. I do not agree that identifying new symptoms will require no adjustments. You want to identify symptoms associated with the diseases but those symptoms could still be subjected to confounders. Some symptoms could as well be due to other underlying health problems associated with upper stream factors, e.g. age, race, and country. Country is a strong one as the health profile between country is different so as the eclampsia incidence.**

A. As requested, we have performed a post-hoc analysis where we adjusted our findings by maternal age, body mass index and country of recruitment. Adjusting for these factors did not materially change the odds ratios (please refer to supplementary table 7, which is a new addition).

We have added the following text:

(In the statistical section in **methods)**

“In a post hoc analysis, multivariable logistic regression was used to estimate adjusted odds ratios, controlling for maternal age, body mass index, and country of recruitment.”

(at the end of **results)**

“We did a second post-hoc analysis to examine whether maternal demographics confounded the associations we report between symptoms and eclampsia. Odds ratios for novel prodromal symptoms and known symptoms did not materially change when we corrected for maternal age, body mass index and country of recruitment (supplementary table 7). The odds ratios for the novel prodromal symptoms ranged between 9.33-40.98 whereas odds ratios for the known symptoms of headache, visual changes and epigastric pain ranged between 1.8-6.3.”

Also note the addition of supplementary table 7.

**Reviewer #2:**

All questions have been answered satisfactorily! No further comments.

**Reviewer #3:**

Thank you for comprehensively addressing all the comments
