# Supplementary material for: Identifying novel prodromal symptoms of eclampsia: A two-country, case-control study
Source: PLoS Med. 2026 Apr 28;23(4):e1004994. doi: 10.1371/journal.pmed.1004994 (PMC13123989; doi:10.1371/journal.pmed.1004994)
Supplement: Attachment — Submitted filename: Rebuttal 3rd resubmission 19 2 26.docx [file pmed.1004994.s011.docx]

Dear Suzanne De Bruijn, Associate Editor of PLOS Medicine,

We thank you for the provisional acceptance of our paper. This project has run for many years and we are delighted.

We have addressed all comments, and item them here in the rebuttal. Similarly to prior submissions, we have uploaded marked and clean copies.

Kind regards

Stephen Tong (corresponding author)

**Editorial requests**

**Please change your title to: “identifying novel prodromal symptoms of eclampsia: a two-country, case-control study ”**

A: Done.

**Please ensure that all abbreviations are defined at first use throughout the text.**

A: Done.

**Please confirm that all numbers presented in the abstract are present and identical to numbers presented in the main manuscript text.**

A: Done.

**Statistical reporting: Please revise throughout the manuscript, including tables and figures.**

A: Done.

**Please report statistical information as follows to improve clarity for the reader ""22% (95% CI [13,28]; p</=)"".**

A: Done.

**Please separate upper and lower bounds with commas instead of hyphens as the latter can be confused with reporting of negative values.**

A: Done.

**Please repeat statistical definitions (HR, CI etc.) for each set of parentheses."**

A: We have defined HR and CI wherever it is used.

**In the last sentence of the Abstract Methods and Findings section, please describe the main limitation(s) of the study's methodology.**

A: Done. We added the following to that section:

“This study is limited by fact we asked about prodromal symptoms after the seizure happened, and the potential for recall bias.”

**Please ensure that the study is reported according to the STROBE guideline, and include the completed STROBE checklist as Supporting Information. Please add the following statement, or similar, to the Methods: ""This study is reported as per the Strengthening the Reporting of Observational Studies in Epidemiology (STROBE) guideline (S1 Checklist)." The STROBE guideline can be found here: http://www.equator-network.org/reporting-guidelines/strobe/ When completing the checklist, please use section and paragraph numbers, rather than page numbers."**

A: Done.

**FUNDING STATEMENT**

**The funding statement should include: specific grant numbers, initials of authors who received each award, URLs to sponsors’ websites. Also, please state whether any sponsors or funders (other than the named authors) played any role in study design, data collection and analysis, the decision to publish, or preparation of the manuscript. If they had no role in the research, include this sentence: “The funders had no role in study design, data collection and analysis, decision to publish, or preparation of the manuscript.”**

A: Done.

**AUTHOR SUMMARY**

**Why was this study done: point 2: Please revise to "Giving magnesium sulphate can half the risk of eclampsia, but predicting who is at risk of an eclamptic seizure is challenging.”**

A: Done.

**"what did the researchers do and find" : there is a typo in ‘Researcher’, please amend.**

A: Done.

**"what did the researchers do and find": please combine point 1 and point 2. We suggest: “We performed a study in Cape Town and Pakistan, in which we asked women who had a recent eclamptic seizure which symptoms they experienced (before the seizure occurred), among a list of novel neurological symptoms, which have not been reported to be associated with eclampsia.’**

A: Done.

**What did the researchers do and find, 5th point: We suggest to remove this, as we limit the number of bullet points to 3.**

A: Done.

**Reviewer 1**

**Thank you for revising the title and conducting the additional analysis – the consistency adds confidence to the findings**

A: Thank you.
